# Surveillance for Violent Deaths — National Violent Death Reporting System, 50 States, the District of Columbia, and Puerto Rico, 2023

**DOI:** 10.15585/mmwr.ss7507a1

**Published:** 2026-10-01

**Authors:** Katherine A. Fowler, Saskia Vos, Kara Tsukerman, Kameron J. Sheats, Carter J. Betz, Kaitlin Forsberg

**Affiliations:** 1 Division of Violence Prevention, National Center for Injury Prevention and Control, CDC

## Abstract

**Problem/Condition:**

In 2023, approximately 23,000 persons died of homicide and 49,000 persons died of suicide in the United States, according to the National Vital Statistics System. This report summarizes data from CDC’s National Violent Death Reporting System (NVDRS) on violent deaths and suicides that occurred in the 50 states, the District of Columbia, and Puerto Rico in 2023.

**Period Covered:**

2023.

**Description of System:**

NVDRS collects data from death certificates, coroner and medical examiner records, and law enforcement reports. This report includes data collected from all 50 states, the District of Columbia, and Puerto Rico. Forty-seven states had statewide data, three additional states had data from counties representing a subset of their population (31 California counties, representing 74.1% of its population; 38 Florida counties, representing 59.3% of its population; and 13 Texas counties, representing 62.9% of its population), and the District of Columbia and Puerto Rico had jurisdiction-wide data. NVDRS collates information for each death and links deaths that are related (e.g., multiple homicides, homicide followed by suicide, or multiple suicides) into a single incident.

**Results:**

For 2023, NVDRS collected information on 70,133 fatal incidents involving 72,093 deaths. The deaths recorded in NVDRS accounted for 91.6% of all homicides, legal intervention deaths, suicides, unintentional firearm injury deaths, and deaths of undetermined intent in the United States in 2023. In addition, information was collected for 671 fatal incidents involving 730 deaths in Puerto Rico. Data for Puerto Rico were analyzed separately.

Of the 72,093 deaths that occurred in the 50 states and the District of Columbia, the majority (62.4%) were suicides, followed by homicides (28.5%), deaths of undetermined intent that might be due to violence (6.9%), legal intervention deaths (1.5%) (i.e., deaths caused by law enforcement and other persons with legal authority to use deadly force acting in the line of duty, excluding legal executions), and unintentional firearm injury deaths (<1.0%). Of the 730 deaths reported for Puerto Rico, 66.4% were homicides and 32.9% were suicides.

Demographic patterns and circumstances varied by manner of death. In the 50 states and the District of Columbia, the suicide rate was higher for males than for females (23.5 versus 6.1 per 100,000 population). The suicide rate for males was highest for those aged ≥85 years (53.0), whereas for females, the suicide rate was highest for those aged 45–54 years (8.8). In addition, non-Hispanic American Indian or Alaska Native (AI/AN) persons had the highest suicide rates among all racial and ethnic groups (22.7). Among both males and females, the most common method of injury for suicide was a firearm. Among all suicide decedents for whom circumstances were known, suicide was most often preceded by an intimate partner, mental health, or physical health problem or by a recent or impending crisis.

The homicide rate was also higher for males (10.8 per 100,000 population) than for females (2.7). Among all homicide victims, the homicide rate was highest among adults aged 20–24 years (14.1) compared with other age groups. Non-Hispanic Black or African American (Black) males experienced the highest homicide rate of any racial or ethnic group (50.6). Among all homicide victims, the most common method of injury was a firearm. When the relationship between a homicide victim and a suspect was known, the suspect was most frequently an acquaintance or friend for male victims and a current or former intimate partner for female victims. When circumstances were known, homicide was most often precipitated by an argument or conflict, occurred in conjunction with another crime, or, for female victims, was related to intimate partner violence. Nearly all victims of legal intervention deaths were male (95.6%) and the legal intervention death rate was highest among men aged 30–34 years. The legal intervention death rate was highest among AI/AN males, followed by Black males. A firearm was used in most legal intervention deaths. When circumstances were known, the most frequently reported circumstances for legal intervention deaths were the victim’s reported use of a weapon, the victim being previously known to authorities, or that the victim reportedly had mental health or substance use problems.

Other manners of death included unintentional firearm injury deaths and deaths of undetermined intent. Unintentional firearm injury deaths occurred most frequently among males, non-Hispanic White (White) persons, and persons aged 15–19 years. When circumstances were known, these deaths most frequently occurred while the shooter was playing with a firearm and most often were precipitated by a person unintentionally pulling the trigger. The rate of deaths of undetermined intent was highest among males, particularly among AI/AN and Black males, and among adults aged 35–44 years. Poisoning was the most common method of injury in deaths of undetermined intent, often involving opioids (67% positive among decedents tested).

In Puerto Rico, the homicide rate was twice as high as the suicide rate and was 10.7 times higher for males than for females. Firearms were the most common method of injury in homicides. When the relationship between the homicide victim and suspect was known, the suspect was most frequently an acquaintance or friend for male victims and a current or former intimate partner for female victims. Among male homicide victims in Puerto Rico when circumstances were known, the most common precipitating circumstances were drug involvement and the victim previously being known to authorities, whereas among female victims, the most common homicide circumstance was intimate partner violence. The suicide rate in Puerto Rico was also higher for males than for females. The most common suicide method was hanging, strangulation, or suffocation. Depressed mood or currently diagnosed mental health problem were frequent circumstances reported for both male and female suicide decedents.

**Interpretation:**

This report summarizes NVDRS data on violent deaths and suicides that occurred in 2023. The suicide rate was highest among AI/AN and White males, whereas the homicide rate was highest among Black males. Intimate partner violence precipitated a large proportion of homicides of females. Mental health and substance use problems, previous awareness of the victim by authorities, intimate partner problems, interpersonal conflicts, and acute life stressors were the primary precipitating circumstances for multiple types of deaths examined.

**Public Health Action:**

The injury-related deaths described in this report are preventable, and data can guide public health action. NVDRS data are used to monitor the occurrence of these fatal injuries and assist public health agencies in developing, implementing, and evaluating programs, policies, and practices to reduce and prevent deaths. Violent death reporting system data are helping states identify disproportionately affected groups and implement targeted prevention strategies, including youth suicide–focused analyses in Maryland and veteran suicide–focused initiatives in Michigan and Connecticut that guided statewide partnerships, crisis line outreach, and mental health service expansion.

## Introduction

According to National Vital Statistics System mortality data obtained from the CDC’s Web-based Injury Statistics Query and Reporting System (WISQARS),* approximately 78,000 deaths in the United States in 2023 were due to suicide, homicide, legal intervention, or unintentional firearm injuries or were deaths of undetermined intent that might have been due to violence (*1*). Public health authorities require accurate, timely, and complete surveillance data to better understand and ultimately prevent violent deaths and suicides in the United States (*2*). CDC’s National Violent Death Reporting System (NVDRS) collects data on suicides, homicides, legal intervention deaths (i.e., deaths caused by law enforcement acting in the line of duty and other persons with legal authority to use deadly force, excluding legal executions), unintentional firearm injury deaths, and deaths of undetermined intent that might have occurred because of violence or suicide.^†^ The term “legal intervention” is a classification incorporated into the *International Classification of Diseases, Tenth Revision* (*ICD-10*) (*3*) and does not denote the lawfulness or legality of the circumstances surrounding a death caused by law enforcement.

NVDRS is a jurisdiction-based active surveillance system that collects data on the characteristics and circumstances associated with violent deaths and suicides in all 50 states, the District of Columbia, and Puerto Rico (*4*). Before the implementation of NVDRS, single data sources (e.g., death certificates) provided only limited information and few circumstances from which to understand patterns of violent deaths and suicides. NVDRS is the first system to 1) provide detailed information on circumstances precipitating violent deaths and suicides, 2) link multiple source documents so that each incident can contribute to the study of patterns of violent deaths and suicides, and 3) link multiple deaths that are related to one another (e.g., multiple homicides, suicide pacts, or homicide followed by suicide of the suspect).

The goals of NVDRS are to

- collect and analyze timely, high-quality data for monitoring the magnitude and characteristics of violent deaths and suicides at national, state, and local levels;
- ensure data are disseminated routinely and expeditiously to public health officials, law enforcement officials, policymakers, and the public;
- ensure data are used to develop, implement, and evaluate programs and strategies that are intended to reduce and prevent violent deaths and suicides at national, state, and local levels; and
- build and strengthen partnerships among organizations and communities at national, state, and local levels to ensure that data are collected and used to reduce and prevent violent deaths and suicides.

This report summarizes NVDRS data on violent deaths and suicides that occurred in the 50 states, the District of Columbia, and Puerto Rico in 2023. New for the 2023 data year, this report has a more concise format, including streamlined or consolidated methodologic and definition sections.

## Methods

NVDRS compiles information from three data sources: death certificates, coroner and medical examiner records, and law enforcement reports (*4*). The ability to analyze linked data can provide a more comprehensive understanding of violent deaths and suicides. Participating VDRS programs use vital statistics death certificate files or coroner or medical examiner records to identify deaths meeting the NVDRS case definition (see Manner of Death). When a case definition matching a violent death or suicide is identified, NVDRS data abstractors link source documents and deaths within each incident, code data elements, and write brief narratives of the incident.

In NVDRS, a case is defined as a death resulting from the intentional use of physical force or power, threatened or actual, against oneself, another person, or a group or community (*4*). NVDRS collects information about homicides, suicides, deaths by legal intervention (excluding legal executions), and deaths of undetermined intent that might have occurred because of violence. NVDRS also collects information on unintentional firearm injury deaths. Deaths resulting from injuries that are clearly linked by source documents and occur within 24 hours of each other are considered part of the same incident. Information collected from each data source is de-identified and entered into the NVDRS web-based system by trained state and jurisdiction-level data abstractors.

Ongoing coding support for data abstractors is provided by CDC through an NVDRS coding manual (*5*), with publicly available CDC-issued standard guidance on coding criteria and examples for each data element provided to each VDRS program. Software features that enhance coding reliability include automated validation rules and a hover-over feature containing variable-specific information. Each year, VDRS programs are required to reabstract at least 5% of cases using multiple abstractors to identify inconsistencies. CDC conducts data quality checks to identify inconsistencies and duplicate cases before data are finalized. This activity was reviewed by CDC, deemed not research, and conducted consistent with applicable federal law and CDC policy.^§^

### Manner of Death

A manner (i.e., intent) of death for each decedent is assigned by a trained abstractor who integrates information from all source documents. The abstractor-assigned manner of death must be consistent with the manner of death listed on at least one required data source (*5*).

NVDRS data are categorized into five abstractor-assigned manners of death: 1) suicide, 2) homicide, 3) legal intervention death, 4) unintentional firearm injury death, and 5) death of undetermined intent, defined as follows:

- **Suicide.** A suicide is a death resulting from the use of force against oneself when a preponderance of evidence indicates that the use of force was intentional. This category does not include deaths caused by chronic or acute substance use without the intent to die, deaths attributed to autoerotic behavior (e.g., self-strangulation during sexual activity), or assisted suicides (legal or nonlegal). Corresponding *ICD-10* codes included in NVDRS are X60–X84, Y87.0, and U03 (*3*).
- **Homicide.** A homicide is a death resulting from the use of physical force or power, threatened or actual, against another person, group, or community when a preponderance of evidence indicates that the use of force was intentional. This category excludes vehicular homicide without intent to injure, unintentional poisoning deaths involving illegal or prescription drugs even when the person who provided drugs was charged with homicide, unintentional firearm injury deaths (a separate category in NVDRS), combat deaths or acts of war, deaths of unborn fetuses, and deaths of infants that resulted indirectly from violence sustained by the mother before birth (e.g., death from prematurity after premature labor brought on by violence). Corresponding *ICD-10* codes included in NVDRS are X85–X99, Y00–Y09, Y87.1, and U01–U02 (*3*).
- **Legal intervention.** A death from legal intervention is a death in which a person is killed or died as a result of injuries inflicted by a law enforcement officer or another peace officer (i.e., a person with specified legal authority to use deadly force), including military police, while acting in the line of duty. The term “legal intervention” is a classification from *ICD-10* (Y35.0) and does not denote the lawfulness or legality of the circumstances surrounding a death caused by law enforcement. Legal intervention deaths also include a small subset of cases in which force was applied without clear lethal intent (e.g., during restraint or when applying force with a typically nondeadly weapon, such as a stun gun (e.g., Taser) or in which the death occurred while the person was fleeing capture. This category excludes legal executions. Corresponding *ICD-10* codes included in NVDRS are Y35.0–Y35.4, Y35.6, Y35.7, and Y89.0 (*3*).
- **Unintentional firearm injury.** An unintentional firearm injury death is a death resulting from a penetrating injury or gunshot wound from a weapon that uses a powder charge to fire a projectile and for which a preponderance of evidence indicates that the shooting was not directed intentionally at the decedent with an intent to injure. This category excludes injuries caused by unintentionally striking a person with the firearm (e.g., hitting a person on the head with the firearm rather than firing a projectile) and unintentional injuries from non-powder guns (e.g., BB, pellet, or other compressed air-powered or compressed gas-powered guns). Corresponding *ICD-10* codes included in NVDRS are W32–W34 and Y86 (*3*).
- **Undetermined intent.** A death of undetermined intent is a death resulting from the use of force or power against oneself or another person for which the evidence indicating one manner of death is no more compelling than evidence indicating another. This category includes coroner or medical examiner rulings in which records from data providers indicate that investigators did not find enough evidence to determine whether the injury was intentional (e.g., unclear whether a drug overdose was unintentional or a suicide). Corresponding *ICD-10* codes included in NVDRS are Y10–Y34, Y87.2, and Y89.9 (*3*).

### Variables Analyzed

NVDRS collects hundreds of unique variables. The number of variables recorded for each incident depends on the content and completeness of the source documents. Variables in NVDRS include

- manner of death (i.e., the intent to cause death [suicide, homicide, legal intervention, unintentional firearm, and undetermined] of the person on whom a fatal injury was inflicted);
- demographic information (e.g., age, sex, and race and ethnicity) of victims and suspects (if applicable);
- method of injury (i.e., the mechanism used to inflict a fatal injury);
- location, date, and time of injury and death;
- toxicology findings for decedents who were tested (positive results included any detectable level of a substance);
- circumstances (i.e., the events that preceded, precipitated, or occurred during or otherwise contributed to the fatal incident as identified by investigators as relevant and therefore might have contributed to the infliction of a fatal injury);
- whether the decedent was a victim (i.e., a person who died as a result of a suicide or violence-related injury) or both a suspect and a victim (i.e., a person believed to have inflicted a fatal injury on a victim who then was fatally injured, such as the perpetrator of a homicide followed by suicide);
- information about any known suspects (i.e., a person or persons believed to have inflicted a fatal injury on a victim);
- incident (i.e., an occurrence in which one or more persons sustained a fatal injury that was linked to a common event or perpetrated by the same suspect or suspects during a 24-hour period); and
- type of incident (i.e., a combination of the manner of death and whether single or multiple victims were involved in an incident).

### Time Frame

VDRS programs are required to begin entering each death into the web-based system within 3 months from the date the death occurred. This report focuses on deaths occurring in 2023. Deaths of multiple-victim incidents are included if the first death of the incident occurred in 2023. VDRS programs have an additional 16 months from the end of the calendar year in which the death occurred to complete each incident record. Although VDRS programs typically meet timeliness requirements, additional details or new incidents occasionally are identified after deadlines and are incorporated on an ongoing basis; 9 months after the 16-month data collection period, systemwide case counts had increased by 2.5% for data year 2023.

### Inclusion Criteria

The inclusion criteria for deaths in this report are as follows: 1) cases met the NVDRS case definition, 2) cases occurred in states and jurisdictions participating in NVDRS in 2023, and 3) each of the included states or jurisdictions had circumstance information from the coroner or medical examiner record or law enforcement report for at least 50% of cases.

Of the participating VDRS programs, 47 states (all except California, Florida, and Texas), the District of Columbia, and Puerto Rico collected information on all violent deaths and suicides that occurred in their state or jurisdiction in 2023. California, Florida, and Texas collected data on deaths in a subset of counties. 

California collected data from death certificates for all violent deaths and suicides in in the state in 2023 (n = 6,718); data for deaths that occurred in 31 counties (Supplementary Box) also included information from coroner or medical examiner records and law enforcement reports and are included throughout the rest of the report (n = 4,728 [70.4% of all cases in the state]). These 31 counties represented 74.1% of California’s population (*6*).

Florida collected data from death certificates for all violent deaths and suicides in the state in 2023 (n = 5,260); data for deaths that occurred in 38 counties (Supplementary Box) also included information from coroner or medical examiner records and law enforcement reports and are included throughout the rest of the report (n = 3,207 [61.0% of all cases in the state]). These 38 counties represented 59.3% of the state’s population (*6*).

Texas collected data from death certificates for all violent deaths and suicides in the state in 2023 (n = 6,982); data for deaths that occurred in 13 counties (Supplementary Box) also included information from coroner or medical examiner records and law enforcement reports and are included throughout the rest of the report (n = 4,235 [60.7% of all cases in the state]). These 13 counties represented 62.9% of the state’s population (*6*).

Data for deaths from California, Florida, and Texas that only had information from death certificates are available (Supplementary Table 1). For all other analyses involving California, Florida, and Texas, only deaths from the counties listed were included. Because <100% of deaths were fully abstracted, data reported in the main tables from California, Florida, and Texas do not represent all cases occurring in these states.

### Analyses

VDRS program–level data received by CDC as of May 24, 2025, were consolidated and analyzed. The numbers, percentages, and crude rates are presented in aggregate for all deaths by the abstractor-assigned manner of death. For each rate, the numerator was the number of deaths in the specified category, and the denominator was the corresponding population estimate; deaths with missing or unknown information for a given characteristic were excluded from the numerator and denominator when applicable. The rates for <20 deaths are not reported because the resulting rates are unstable. Denominators for the rates for the three states that did not collect statewide data (California, Florida, and Texas) correspond to the populations of the counties from which data were collected. The rates could not be calculated for certain variables (e.g., circumstances) because denominators were unknown.

The U.S. Census Bureau’s county-level population estimates for 2023 were used as denominators in the crude rate calculations for the 50 states (47 states collecting statewide data, 31 California counties, 38 Florida counties, and 13 Texas counties) and the District of Columbia (*7*). Data for Puerto Rico were analyzed separately: the rates specific to race and ethnicity are not available for Puerto Rico because the U.S. Census Bureau estimates for Puerto Rico do not include race or Hispanic or Latino (Hispanic) origin (*8*). Population estimates by sex and age were used as denominators in the crude rate calculations for Puerto Rico (*9*).

## Results

### Deaths in the 50 States and the District of Columbia

For 2023, all 50 U.S. states (47 states collecting statewide data, 31 California counties, 38 Florida counties, and 13 Texas counties) and the District of Columbia collected data on 70,133 incidents involving 72,093 deaths (Supplementary Table 2). Suicides (n = 44,966 across 44,937 incidents [62.4% of all deaths]) accounted for the highest rate of deaths recorded by NVDRS (14.7 per 100,000 population). The homicide rate was 6.7 per 100,000 population (n = 20,540 [28.5%]). Deaths of undetermined intent (n = 4,962 [6.9%]), legal intervention deaths (n = 1,083 [1.5%]), and unintentional firearm injury deaths (n = 542; [<1.0%]) occurred at lower rates (1.6, 0.4, and 0.2, respectively). Data for deaths by manner that include statewide counts and the rates for California, Florida, and Texas are available (Supplementary Table 1). More than half of NVDRS deaths (58.6%) involved firearms as the method of injury, and the majority of victims (62.3%) were injured in a house or apartment (Supplementary Table 3).

### Suicides

#### Demographics

The overall suicide rate for males (23.5) was nearly 4 times the rate for females (6.1) (Table 1). The suicide rate for males ranged from 1.2 to 11.5 times the rate for females across age groups and 2.6 to 4.3 times the rate for females across racial and ethnic groups. Adults aged ≥85 years (22.4), 75–84 years (19.2), and 35–44 years (19.1) had the highest rates of suicide across age groups. White persons accounted for most (76.2%) suicides; however, non-Hispanic American Indian or Alaska Native (AI/AN) persons had the highest rate of suicide (22.7) among all racial and ethnic groups.

**TABLE 1:** Number, percentage,* and rate^†^ of suicides, by selected demographics of decedent,^§^ method of injury, location of injury, and incident characteristics — National Violent Death Reporting System, 50 states^¶^ and District of Columbia, 2023

Characteristic	Female		Male		Total	
	No. (%)	Rate	No. (%)	Rate	No. (%)	Rate
**Age group, yrs**						
<10	2 (<1.0)	—**	3 (<1.0)	—	**5 (<1.0)**	**—**
10–14	190 (2.0)	2.1	243 (<1.0)	2.5	**433 (<1.0)**	**2.3**
15–19	477 (5.1)	4.9	1,493 (4.2)	14.5	**1,970 (4.4)**	**9.8**
20–24	620 (6.6)	6.3	2,856 (8.0)	28.0	**3,477 (7.7)**	**17.4**
25–29	721 (7.6)	7.2	2,987 (8.4)	29.0	**3,710 (8.3)**	**18.3**
30–34	825 (8.7)	7.7	3,167 (8.9)	29.0	**3,995 (8.9)**	**18.5**
35–44	1,626 (17.2)	8.1	6,150 (17.3)	30.1	**7,777 (17.3)**	**19.1**
45–54	1,634 (17.3)	8.8	5,345 (15.0)	29.1	**6,979 (15.5)**	**18.9**
55–64	1,650 (17.5)	8.5	5,512 (15.5)	29.4	**7,162 (15.9)**	**18.7**
65–74	1,048 (11.1)	6.2	3,925 (11.0)	26.3	**4,973 (11.1)**	**15.7**
75–84	476 (5.0)	5.1	2,733 (7.7)	37.0	**3,209 (7.1)**	**19.2**
≥85	163 (1.7)	4.6	1,108 (3.1)	53.0	**1,271 (2.8)**	**22.4**
Unknown	1 (<1.0)	—	4 (<1.0)	—	**5 (<1.0)**	**—**
**Race and ethnicity^††^**						
American Indian or Alaska Native	134 (1.4)	11.4	392 (1.1)	34.3	**526 (1.2)**	**22.7**
Asian	368 (3.9)	3.7	894 (2.5)	9.7	**1,262 (2.8)**	**6.6**
Black or African American	731 (7.7)	3.6	2,910 (8.2)	15.4	**3,641 (8.1)**	**9.2**
Hispanic or Latino	809 (8.6)	3.0	3,504 (9.9)	12.8	**4,313 (9.6)**	**8.0**
Native Hawaiian or Pacific Islander	18 (<1.0)	—	78 (<1.0)	25.5	**96 (<1.0)**	**15.8**
White	7,154 (75.8)	7.8	27,094 (76.3)	29.9	**34,254 (76.2)**	**18.7**
More than one race	162 (1.7)	4.2	448 (1.3)	11.9	**611 (1.4)**	**8.0**
Unspecified or unknown	57 (<1.0)	—	206 (<1.0)	—	**263 (<1.0)**	**—**
**Method of injury**						
Firearm	3,222 (34.2)	2.1	21,294 (59.9)	14.1	**24,516 (54.5)**	**8.0**
Hanging, strangulation, or suffocation	2,532 (26.8)	1.6	8,680 (24.4)	5.7	**11,215 (24.9)**	**3.7**
Poisoning	2,686 (28.5)	1.7	2,309 (6.5)	1.5	**4,997 (11.1)**	**1.6**
Fall	316 (3.3)	0.2	900 (2.5)	0.6	**1,217 (2.7)**	**0.4**
Sharp instrument	167 (1.8)	0.1	815 (2.3)	0.5	**982 (2.2)**	**0.3**
Motor vehicle (e.g., bus, motorcycle, or other transport vehicle)	188 (2.0)	0.1	534 (1.5)	0.4	**722 (1.6)**	**0.2**
Drowning	114 (1.2)	<0.1	260 (<1.0)	0.2	**374 (<1.0)**	**0.1**
Fire or burns	47 (<1.0)	<0.1	112 (<1.0)	<0.1	**159 (<1.0)**	**<0.1**
Blunt instrument	22 (<1.0)	<0.1	50 (<1.0)	<0.1	**72 (<1.0)**	**<0.1**
Other (e.g., Taser, electrocution, nail gun, intentional neglect, or personal weapon)	12 (<1.0)	—	54 (<1.0)	—	**66 (<1.0)**	**—**
Unknown	127 (1.3)	—	518 (1.5)	—	**646 (1.4)**	**—**
**Location of injury**						
House or apartment	7,306 (77.5)	4.7	24,968 (70.3)	16.5	**32,280 (71.8)**	**10.5**
Motor vehicle	440 (4.7)	0.3	2,202 (6.2)	1.5	**2,642 (5.9)**	**0.9**
Natural area	309 (3.3)	0.2	1,743 (4.9)	1.2	**2,052 (4.6)**	**0.7**
Street or highway	181 (1.9)	0.1	992 (2.8)	0.7	**1,174 (2.6)**	**0.4**
Hotel or motel	269 (2.9)	0.2	783 (2.2)	0.5	**1,052 (2.3)**	**0.3**
Parking lot, public garage, or public transport	96 (1.0)	<0.1	608 (1.7)	0.4	**704 (1.6)**	**0.2**
Park, playground, or sports or athletic area	90 (<1.0)	<0.1	605 (1.7)	0.4	**695 (1.5)**	**0.2**
Jail or prison	45 (<1.0)	<0.1	541 (1.5)	0.4	**586 (1.3)**	**0.2**
Commercial or retail area	47 (<1.0)	<0.1	415 (1.2)	0.3	**462 (1.0)**	**0.2**
Bridge	71 (<1.0)	<0.1	286 (<1.0)	0.2	**357 (<1.0)**	**0.1**
Railroad tracks	68 (<1.0)	<0.1	220 (<1.0)	0.1	**288 (<1.0)**	**<0.1**
Other location^§§^	331 (3.5)	—	1,437 (4.0)	—	**1,768 (3.9)**	**—**
Unknown	180 (1.9)	—	726 (2.0)	—	**906 (2.0)**	**—**
**Incident characteristic**						
Emergency medical services present	6,755 (71.6)	4.4	23,925 (67.3)	15.8	**30,684 (68.2)**	**10.0**
Injured at victim’s home	6,723 (71.3)	4.3	22,637 (63.7)	15.0	**29,364 (65.3)**	**9.6**
Victim was suspected of alcohol use preceding the incident	1,360 (14.4)	0.9	5,370 (15.1)	3.5	**6,732 (15.0)**	**2.2**
Victim was recently released from an institutional setting	611 (6.5)	0.4	1,889 (5.3)	1.2	**2,500 (5.6)**	**0.8**
Child present or witnessed incident	539 (5.7)	0.3	1,536 (4.3)	1.0	**2,075 (4.6)**	**0.7**
Victim was experiencing housing instability	241 (2.6)	0.2	899 (2.5)	0.6	**1,140 (2.5)**	**0.4**
Victim was in public custody when injury occurred	86 (<1.0)	<0.1	999 (2.8)	0.7	**1,085 (2.4)**	**0.4**
Victim was experiencing homelessness	116 (1.2)	<0.1	582 (1.6)	0.4	**698 (1.6)**	**0.2**
Victim was injured at work or while working	24 (<1.0)	<0.1	367 (1.0)	0.2	**391 (<1.0)**	**0.1**
**Total**	**9,433 (100)**	**6.1**	**35,526 (100)**	**23.5**	**44,966 (100)**	**14.7**

* Percentages might not total 100% due to rounding.

^†^ Per 100,000 population.

^§^ Sex was unknown for seven decedents.

^¶^ Data for California are for deaths that occurred in 31 counties (Alameda, Alpine, Amador, Butte, Fresno, Humboldt, Imperial, Kings, Lake, Lassen, Los Angeles, Madera, Mendocino, Merced, Mono, Orange, Placer, Riverside, Sacramento, San Benito, San Diego, San Francisco, San Luis Obispo, San Mateo, Santa Cruz, Shasta, Solano, Sonoma, Tehama, Ventura, and Yolo), data for Florida are for deaths that occurred in 38 counties (Alachua, Bay, Brevard, Calhoun, Charlotte, Citrus, Escambia, Flagler, Gilchrist, Hardee, Hernando, Highlands, Hillsborough, Holmes, Indian River, Jackson, Lake, Levy, Manatee, Marion, Martin, Okaloosa, Okeechobee, Orange, Osceola, Palm Beach, Pinellas, Polk, Putnam, St. Johns, St. Lucie, Santa Rosa, Seminole, Sumter, Union, Volusia, Walton, and Washington), and data for Texas are for deaths that occurred in 13 counties (Bell, Bexar, Collin, Dallas, Denton, El Paso, Fort Bend, Harris, Montgomery, Nueces, Tarrant, Travis, and Williamson). Denominators for the rates for California, Florida, and Texas represent only the populations of the counties from which the data were collected.

** Dashes indicate cell data are suppressed because number of decedents is <20 or characteristic response is “other” or “unknown.”

^††^ Persons of Hispanic or Latino (Hispanic) origin might be of any race but are categorized as Hispanic; all racial groups are non-Hispanic.

^§§^ Other location includes (in descending order): supervised residential facility; hospital or medical facility; industrial or construction area; cemetery, graveyard, or other burial ground; preschool, school, college, or school bus; farm; office building; abandoned house, building, or warehouse; synagogue, church, or temple; bar or nightclub; and other unspecified location.

By age group and sex, men aged ≥85 years had the highest rate of suicide (53.0 per 100,000 population), followed by men aged 75–84 years (37.0), 35–44 years (30.1), and 55–64 years (29.4) (Table 1). Across racial and ethnic groups, AI/AN males had the highest rate of suicide (34.3), followed by White males (29.9) and Native Hawaiian or Pacific Islander (NH/PI) males (25.5). The rate of suicide for AI/AN males was 3.5 times the rate for males with the lowest rate (non-Hispanic Asian [Asian]; 9.7). The suicide rate was 15.4 for Black or African American (Black) males, 11.9 for males of more than one race, and 12.8 for Hispanic males.

Among females, those women aged 45–54 years had the highest rate of suicide (8.8 per 100,000 population), followed by those aged 55–64 years (8.5), and 35–44 years (8.1) (Table 1). The suicide rate was highest among AI/AN females (11.4), followed by White females (7.8), females of more than one race (4.2), and Asian females (3.7). The suicide rate for AI/AN females was 3.8 times the rate for females with the lowest rate (Hispanic; 3.0).

#### Method and Location of Injury

A firearm was used in approximately one half (54.5% [8.0 per 100,000 population]) of suicides, followed by hanging, strangulation, or suffocation (24.9% [3.7]) and poisoning (11.1% [1.6]) (Table 1). Among males, the most common method of injury was a firearm (59.9%), followed by hanging, strangulation, or suffocation (24.4%). Among females, a firearm (34.2%) was also the most common method of injury, followed by poisoning (28.5%) and hanging, strangulation, or suffocation (26.8%). Among all suicide decedents, the most common location of suicide was a house or apartment (71.8%), followed by a motor vehicle (5.9%), a natural area (4.6%), a street or highway (2.6%), and a hotel or motel (2.3%).

#### Incident Characteristics

Emergency medical services responded to the scene for a large percentage of suicide decedents (68.2%) (Table 1). Suicide decedents were commonly fatally injured in their own homes (65.3%). Male and female suicide decedents had similar percentages of suspected alcohol use at the time of their death (15.1% and 14.4%, respectively). A child was either present or witnessed the incident for 4.6% of suicide decedents. A small proportion of suicide decedents were experiencing housing instability (2.5%) or homelessness (1.6%) at the time of death or had been recently released from an institutional setting (5.6%).

#### Toxicology Results of Decedent

Toxicology tests for blood alcohol concentration (BAC) were conducted for 50.3% of suicide decedents (Table 2). Among those tested, 38.4% had positive test results for alcohol (i.e., ethanol), and 64.7% had a BAC ≥0.08 g/dL. The proportion of decedents who received testing for a substance varied, and among those tested, the positive results differed by substance, as follows: amphetamines (41.2% tested, of which 15.4% were positive), anticonvulsants (26.2% tested, of which 15.6% were positive), antidepressants (29.6% tested, of which 32.2% were positive), antipsychotics (24.4% tested, of which 10.4% were positive), barbiturates (37.9% tested, of which 1.6% were positive), benzodiazepines (41.2% tested, of which 18.5% were positive), cannabis (commonly referred to as marijuana; 39.7% tested, of which 27.3% were positive), cocaine (40.3% tested, of which 7.3% were positive), muscle relaxants (26.1% tested, of which 4.7% were positive) and opioids (including illicit and prescription; 42.1% tested, of which 20.9% were positive). Carbon monoxide was tested for a substantially smaller proportion of decedents (8.8%) and was identified in 14.8% of those decedents.

**TABLE 2:** Number* and percentage of suicide decedents tested for alcohol and drugs and whose results were positive,^†^ by toxicology variable — National Violent Death Reporting System, 50 states^§^ and District of Columbia, 2023

Toxicology variable	Tested	Positive
	No. (%)	No. (%)
Blood alcohol concentration^¶^	22,639 (50.3)	8,702 (38.4)
Blood alcohol <0.08 g/dL		2,549 (29.3)
Blood alcohol ≥0.08 g/dL		5,626 (64.7)
Blood alcohol positive, level unknown		527 (6.1)
Amphetamines	18,531 (41.2)	2,850 (15.4)
Anticonvulsants	11,802 (26.2)	1,842 (15.6)
Antidepressants	13,325 (29.6)	4,291 (32.2)
Antipsychotics	10,980 (24.4)	1,137 (10.4)
Barbiturates	17,033 (37.9)	269 (1.6)
Benzodiazepines	18,525 (41.2)	3,427 (18.5)
Cannabis**	17,841 (39.7)	4,869 (27.3)
Carbon monoxide	3,978 (8.8)	587 (14.8)
Cocaine	18,124 (40.3)	1,327 (7.3)
Muscle relaxants	11,748 (26.1)	556 (4.7)
Opioids	18,941 (42.1)	3,950 (20.9)
Other drugs or substances^††^	3,338 (7.4)	3,244 (97.2)

* Number of suicide decedents = 44,966. More than one substance could have been tested for or positive per decedent.

^†^ Percentage is of decedents who received toxicology testing. Denominator for the percentage positive is the number of decedents tested.

^§^ Data for California are for deaths that occurred in 31 counties (Alameda, Alpine, Amador, Butte, Fresno, Humboldt, Imperial, Kings, Lake, Lassen, Los Angeles, Madera, Mendocino, Merced, Mono, Orange, Placer, Riverside, Sacramento, San Benito, San Diego, San Francisco, San Luis Obispo, San Mateo, Santa Cruz, Shasta, Solano, Sonoma, Tehama, Ventura, and Yolo), data for Florida are for deaths that occurred in 38 counties (Alachua, Bay, Brevard, Calhoun, Charlotte, Citrus, Escambia, Flagler, Gilchrist, Hardee, Hernando, Highlands, Hillsborough, Holmes, Indian River, Jackson, Lake, Levy, Manatee, Marion, Martin, Okaloosa, Okeechobee, Orange, Osceola, Palm Beach, Pinellas, Polk, Putnam, St. Johns, St. Lucie, Santa Rosa, Seminole, Sumter, Union, Volusia, Walton, and Washington), and data for Texas are for deaths that occurred in 13 counties (Bell, Bexar, Collin, Dallas, Denton, El Paso, Fort Bend, Harris, Montgomery, Nueces, Tarrant, Travis, and Williamson).

^¶^ Blood alcohol concentration of ≥0.08 g/dL is over the legal limit in all states and is used as the standard for intoxication.

** Commonly referred to as marijuana.

^††^ Other drugs or substances indicated if any results were positive.

#### Precipitating Circumstances

Circumstances from coroner or medical examiner records or law enforcement reports were identified in 38,329 (85.2%) suicides (Table 3). Among decedents who had known circumstances, a current diagnosed mental health problem was the most common circumstance identified, with approximately half (50.3%) of decedents having a current diagnosed mental health problem and 25.9% experiencing a depressed mood at the time of death. Among the 19,285 decedents with a current diagnosed mental health problem, depression or dysthymia (72.2%), an anxiety disorder (25.4%), and bipolar disorder (14.2%) were the most common diagnoses. Alcohol use problems were reported for 18.2% of suicide decedents, and other substance use problems (excluding alcohol) were reported for 16.0% of suicide decedents. Among suicide decedents with circumstance data, 22.3% were receiving mental health or substance use treatment at the time of death and 31.9% had a history of having been treated for a mental health or substance use problem (Table 3).

**TABLE 3:** Number* and percentage^†^ of suicides, by decedent sex and precipitating circumstances — National Violent Death Reporting System, 50 states^§^ and District of Columbia, 2023

Precipitating circumstance	Female	Male	Total
	No. (%)	No. (%)	No. (%)
**Mental health and substance use**			
Current diagnosed mental health problem^¶^	5,398 (65.5)	13,885 (46.2)	**19,285 (50.3)**
Depression or dysthymia	4,070 (75.4)	9,860 (71.0)	**13,932 (72.2)**
Anxiety disorder	1,669 (30.9)	3,235 (23.3)	**4,904 (25.4)**
Bipolar disorder	1,020 (18.9)	1,727 (12.4)	**2,747 (14.2)**
Schizophrenia	333 (6.2)	1,022 (7.4)	**1,356 (7.0)**
Posttraumatic stress disorder	245 (4.5)	918 (6.6)	**1,163 (6.0)**
Attention deficit disorder or attention hyperactivity disorder	170 (3.1)	608 (4.4)	**778 (4.0)**
Dementia	70 (1.3)	280 (2.0)	**350 (1.8)**
Autism spectrum	22 (<1.0)	163 (1.2)	**185 (<1.0)**
Obsessive compulsive disorder	31 (<1.0)	71 (<1.0)	**102 (<1.0)**
Eating disorder	28 (<1.0)	14 (<1.0)	**42 (<1.0)**
Fetal alcohol syndrome	0 (—)	8 (<1.0)	**8 (<1.0)**
Down syndrome	1 (<1.0)	0 (—)	**1 (<1.0)**
Other	211 (3.9)	615 (4.4)	**826 (4.3)**
Unknown	361 (6.7)	1,093 (7.9)	**1,454 (7.5)**
History of ever being treated for a mental health or substance use problem	3,768 (45.7)	8,474 (28.2)	**12,244 (31.9)**
Current depressed mood	2,163 (26.2)	7,755 (25.8)	**9,920 (25.9)**
Current mental health or substance use treatment	2,845 (34.5)	5,691 (18.9)	**8,538 (22.3)**
Alcohol problem	1,312 (15.9)	5,663 (18.8)	**6,976 (18.2)**
Substance use problem (excludes alcohol)	1,425 (17.3)	4,710 (15.7)	**6,136 (16.0)**
Nonadherence to mental health or substance use treatment	500 (6.1)	1,362 (4.5)	**1,863 (4.9)**
Other addiction (e.g., gambling or sex)	50 (<1.0)	222 (<1.0)	**272 (<1.0)**
**Interpersonal factor**			
Intimate partner problem	1,792 (21.7)	7,747 (25.8)	**9,539 (24.9)**
Family relationship problem	696 (8.4)	1,964 (6.5)	**2,660 (6.9)**
Other death (not suicide) of family member or friend	580 (7.0)	1,822 (6.1)	**2,402 (6.3)**
Suicide of family member or friend	217 (2.6)	626 (2.1)	**843 (2.2)**
Other relationship problem (nonintimate and nonfamily)	186 (2.3)	588 (2.0)	**774 (2.0)**
Perpetrator of interpersonal violence during past month**	55 (<1.0)	661 (2.2)	**716 (1.9)**
Victim of interpersonal violence during past month**	70 (<1.0)	68 (<1.0)	**138 (<1.0)**
**Life stressor**			
Crisis during previous or upcoming 2 weeks	2,299 (27.9)	9,702 (32.3)	**12,002 (31.3)**
Physical health problem	1,565 (19.0)	6,061 (20.1)	**7,626 (19.9)**
Argument or conflict	1,219 (14.8)	4,630 (15.4)	**5,850 (15.3)**
Victim known to authorities	1,075 (13.0)	4,648 (15.5)	**5,723 (14.9)**
Job problem	452 (5.5)	2,586 (8.6)	**3,039 (7.9)**
Financial problem	499 (6.1)	2,219 (7.4)	**2,718 (7.1)**
Contributing recent criminal legal problem	195 (2.4)	2,231 (7.4)	**2,426 (6.3)**
Household had contact with local authorities	343 (4.2)	945 (3.1)	**1,288 (3.4)**
Family stressor	320 (3.9)	907 (3.0)	**1,227 (3.2)**
Eviction or loss of home	233 (2.8)	952 (3.2)	**1,185 (3.1)**
Noncriminal legal problem	221 (2.7)	895 (3.0)	**1,116 (2.9)**
School problem	135 (1.6)	300 (<1.0)	**436 (1.1)**
History of traumatic brain injury	66 (<1.0)	356 (1.2)	**422 (1.1)**
History of child abuse or neglect	165 (2.0)	254 (<1.0)	**419 (1.1)**
Physical fight (two persons, not a brawl)	40 (<1.0)	285 (<1.0)	**325 (<1.0)**
Living transition or loss of independent living	55 (<1.0)	270 (<1.0)	**325 (<1.0)**
Traumatic anniversary	74 (<1.0)	193 (<1.0)	**267 (<1.0)**
Caregiver stress	60 (<1.0)	194 (<1.0)	**254 (<1.0)**
Exposure to disaster	50 (<1.0)	186 (<1.0)	**236 (<1.0)**
Caretaker abuse or neglect	33 (<1.0)	59 (<1.0)	**92 (<1.0)**
**Crime and criminal activity**			
Precipitated by another crime	100 (1.2)	1,333 (4.4)	**1,433 (3.7)**
Crime in progress^††^	28 (28.0)	447 (33.5)	**475 (33.1)**
**Suicide and self-harm events**			
History of suicidal thoughts or plans	3,366 (40.8)	9,897 (32.9)	**13,265 (34.6)**
Left a suicide note	3,079 (37.3)	8,473 (28.2)	**11,554 (30.1)**
History of attempting suicide	2,456 (29.8)	4,373 (14.5)	**6,829 (17.8)**
History of nonsuicidal self-harm	555 (6.7)	656 (2.2)	**1,212 (3.2)**
**Suicide disclosure**			
Disclosed suicidal intent	1,738 (21.1)	6,229 (20.7)	**7,967 (20.8)**
Disclosed intent to whom^§§^			
Former or current intimate partner	621 (35.7)	2,390 (38.4)	**3,011 (37.8)**
Family member (excludes intimate partner)	664 (38.2)	2,305 (37.0)	**2,969 (37.3)**
Friend or colleague	284 (16.3)	825 (13.2)	**1,109 (13.9)**
Health care worker	106 (6.1)	322 (5.2)	**428 (5.4)**
Through social media or other electronic means	84 (4.8)	294 (4.7)	**378 (4.7)**
Neighbor	31 (1.8)	123 (2.0)	**154 (1.9)**
Other	120 (6.9)	607 (9.7)	**727 (9.1)**
Unknown	144 (8.3)	480 (7.7)	**624 (7.8)**
**Child decedent incident^¶¶^**			
Previous Child Protective Services report on victim's household	18 (4.4)	18 (2.2)	**36 (3.0)**
Substance use in child victim's household	3 (<1.0)	2 (<1.0)	**5 (<1.0)**
Total***	**8,245 (87.4)**	**30,080 (84.7)**	**38,329 (85.2)**

* Includes suicides with one or more precipitating circumstances. More than one circumstance could have been present per decedent.

^†^ Denominator includes those suicides with one or more precipitating circumstances. The sums of percentages in columns exceed 100% because more than one circumstance could have been present per decedent.

^§^ Data for California are for deaths that occurred in 31 counties (Alameda, Alpine, Amador, Butte, Fresno, Humboldt, Imperial, Kings, Lake, Lassen, Los Angeles, Madera, Mendocino, Merced, Mono, Orange, Placer, Riverside, Sacramento, San Benito, San Diego, San Francisco, San Luis Obispo, San Mateo, Santa Cruz, Shasta, Solano, Sonoma, Tehama, Ventura, and Yolo), data for Florida are for deaths that occurred in 38 counties (Alachua, Bay, Brevard, Calhoun, Charlotte, Citrus, Escambia, Flagler, Gilchrist, Hardee, Hernando, Highlands, Hillsborough, Holmes, Indian River, Jackson, Lake, Levy, Manatee, Marion, Martin, Okaloosa, Okeechobee, Orange, Osceola, Palm Beach, Pinellas, Polk, Putnam, St. Johns, St. Lucie, Santa Rosa, Seminole, Sumter, Union, Volusia, Walton, and Washington), and data for Texas are for deaths that occurred in 13 counties (Bell, Bexar, Collin, Dallas, Denton, El Paso, Fort Bend, Harris, Montgomery, Nueces, Tarrant, Travis, and Williamson).

^¶^ Includes decedents with one or more diagnosed current mental health problems; therefore, sums of percentages for the diagnosed conditions exceed 100%. Denominators for the diagnosed conditions include the number of decedents with one or more current diagnosed mental health problems.

** Interpersonal violence includes but is not limited to intimate partner violence.

^††^ Denominator includes those decedents involved in an incident that was precipitated by another crime.

^§§^ Denominator includes decedents who disclosed intent. The sum of percentages exceeds 100% because more than one response could have been present per decedent.

^¶¶^ Circumstance variables in this category are applicable exclusively to children. Denominator includes decedents aged <18 years for whom circumstances are known (n = 1,212; 807 males and 405 females). Circumstances were unknown for 235 child decedents; total number of child suicide decedents = 1,447.

*** Circumstances were unknown for 6,637 decedents (5,446 males, 1,188 females, and three unknown); total number of suicide decedents = 44,966 (35,526 males, 9,433 females, and seven unknown).

The most commonly reported interpersonal or life stressor–related precipitating circumstances for suicide were a recent or impending crisis during the previous or upcoming 2 weeks (31.3%), an intimate partner problem (24.9%), a physical health problem (19.9%), and an argument or conflict (15.3%) (Table 3) (Supplementary Table 4). Additional circumstances related to suicide included the following: 34.6% of decedents had a history of suicidal thoughts or plans, 30.1% left a suicide note, 20.8% had disclosed suicidal intent to another person, and 17.8% had a history of attempting suicide. Among those who disclosed intent, the greatest proportion of disclosures were to a former or current intimate partner (37.8%), followed by a family member other than an intimate partner (37.3%) and a friend or colleague (13.9%).

When examining known circumstances by sex, a larger percentage of female decedents (65.5%) had a current diagnosed mental health problem than did male decedents (46.2%) (Table 3). Female and male suicide decedents had similar percentages of depressed mood at the time of their death (26.2% and 25.8%, respectively). A larger percentage of female decedents (34.5%) than male decedents (18.9%) were known to have been receiving mental health or substance treatment at the time of death. Suicide events including leaving a suicide note, history of suicidal thoughts or plans, history of attempting suicide, and history of nonsuicidal self-harm occurred more frequently among females than males.

Known circumstances were identified in 1,212 (83.8%) suicides of children and adolescents aged <18 years (Table 3). With respect to two circumstances that only applied to child and adolescent decedents, previous Child Protective Services reports were more frequently reported in female decedents’ households compared with male decedents’ households (4.4% and 2.2%, respectively), and both male and female child suicide decedents had similar percentages of substance use problems reported in their household (both <1.0%).

### Homicides

#### Demographics

For 2023, NVDRS collected data on 19,466 incidents involving one or more homicides (Supplementary Table 2) representing 20,540 homicide deaths. The overall homicide rate was 6.7 per 100,000 population (Table 4).

**TABLE 4:** Number, percentage,* and rate^†^ of homicides, by selected demographics of decedent,^§^ method of injury, location of injury, incident characteristics, and victim-suspect relationship^¶^ — National Violent Death Reporting System, 50 states** and District of Columbia, 2023

Characteristic	Female		Male		Total	
	No. (%)	Rate	No. (%)	Rate	No. (%)	Rate
**Age group, yrs**						
<1	92 (2.2)	5.7	133 (<1.0)	7.9	**225 (1.1)**	**6.8**
1–4	107 (2.6)	1.6	145 (<1.0)	2.1	**252 (1.2)**	**1.9**
5–9	66 (1.6)	0.7	96 (<1.0)	1.0	**162 (<1.0)**	**0.9**
10–14	77 (1.9)	0.8	218 (1.3)	2.3	**295 (1.4)**	**1.6**
15–19	308 (7.5)	3.1	2,053 (12.5)	19.9	**2,361 (11.5)**	**11.7**
20–24	439 (10.6)	4.5	2,380 (14.5)	23.4	**2,819 (13.7)**	**14.1**
25–29	404 (9.8)	4.0	2,134 (13.0)	20.7	**2,538 (12.4)**	**12.5**
30–34	464 (11.2)	4.3	2,265 (13.8)	20.7	**2,729 (13.3)**	**12.6**
35–44	774 (18.7)	3.8	3,209 (19.6)	15.7	**3,983 (19.4)**	**9.8**
45–54	509 (12.3)	2.7	1,803 (11.0)	9.8	**2,312 (11.3)**	**6.3**
55–64	416 (10.1)	2.1	1,218 (7.4)	6.5	**1,634 (8.0)**	**4.3**
65–74	263 (6.4)	1.6	544 (3.3)	3.6	**807 (3.9)**	**2.5**
75–84	155 (3.8)	1.7	169 (1.0)	2.3	**324 (1.6)**	**1.9**
≥85	56 (1.4)	1.6	38 (<1.0)	1.8	**94 (<1.0)**	**1.7**
Unknown	0 (—)	—^††^	5 (<1.0)	—	**5 (<1.0)**	**—**
**Race and ethnicity^§§^**						
American Indian or Alaska Native	59 (1.4)	5.0	225 (1.4)	19.7	**284 (1.4)**	**12.3**
Asian	121 (2.9)	1.2	166 (1.0)	1.8	**287 (1.4)**	**1.5**
Black or African American	1,642 (39.8)	8.0	9,549 (58.2)	50.6	**11,191 (54.5)**	**28.4**
Hispanic or Latino	641 (15.5)	2.4	2,916 (17.8)	10.7	**3,557 (17.3)**	**6.6**
Native Hawaiian or Pacific Islander	8 (<1.0)	—	34 (<1.0)	11.1	**42 (<1.0)**	**6.9**
White	1,542 (37.3)	1.7	3,206 (19.5)	3.5	**4,748 (23.1)**	**2.6**
More than one race	90 (2.2)	2.3	209 (1.3)	5.5	**299 (1.5)**	**3.9**
Unspecified or unknown	27 (<1.0)	—	105 (<1.0)	—	**132 (<1.0)**	**—**
**Method of injury**						
Firearm	2,689 (65.1)	1.7	13,292 (81.0)	8.8	**15,981 (77.8)**	**5.2**
Sharp instrument	503 (12.2)	0.3	1,287 (7.8)	0.9	**1,790 (8.7)**	**0.6**
Blunt instrument	214 (5.2)	0.1	481 (2.9)	0.3	**695 (3.4)**	**0.2**
Personal weapon (e.g., hands, fists, or feet)	158 (3.8)	0.1	432 (2.6)	0.3	**590 (2.9)**	**0.2**
Hanging, strangulation, or suffocation	209 (5.1)	0.1	164 (<1.0)	0.1	**373 (1.8)**	**0.1**
Motor vehicle (e.g., bus, motorcycle, or other transport vehicle)	44 (1.1)	<0.1	124 (<1.0)	<0.1	**168 (<1.0)**	**<0.1**
Poisoning	45 (1.1)	<0.1	83 (<1.0)	<0.1	**128 (<1.0)**	**<0.1**
Fire or burns	39 (<1.0)	<0.1	56 (<1.0)	<0.1	**95 (<1.0)**	**<0.1**
Intentional neglect	37 (<1.0)	<0.1	39 (<1.0)	<0.1	**76 (<1.0)**	**<0.1**
Shaking (e.g., shaken baby syndrome)	10 (<1.0)	—	27 (<1.0)	<0.1	**37 (<1.0)**	**<0.1**
Fall	12 (<1.0)	—	21 (<1.0)	<0.1	**33 (<1.0)**	**<0.1**
Drowning	9 (<1.0)	—	13 (<1.0)	—	**22 (<1.0)**	**<0.1**
Other (e.g., Taser, electrocution, or nail gun)	13 (<1.0)	—	32 (<1.0)	—	**45 (<1.0)**	**—**
Unknown	148 (3.6)	—	359 (2.2)	—	**507 (2.5)**	**—**
**Location of injury**						
House or apartment	2,628 (63.6)	1.7	6,291 (38.3)	4.2	**8,919 (43.4)**	**2.9**
Street or highway	347 (8.4)	0.2	3,791 (23.1)	2.5	**4,138 (20.1)**	**1.4**
Motor vehicle	367 (8.9)	0.2	1,669 (10.2)	1.1	**2,036 (9.9)**	**0.7**
Commercial or retail area	115 (2.8)	<0.1	1,052 (6.4)	0.7	**1,167 (5.7)**	**0.4**
Parking lot, public garage, or public transport	100 (2.4)	<0.1	814 (5.0)	0.5	**914 (4.4)**	**0.3**
Natural area	91 (2.2)	<0.1	278 (1.7)	0.2	**369 (1.8)**	**0.1**
Bar or nightclub	29 (<1.0)	<0.1	299 (1.8)	0.2	**328 (1.6)**	**0.1**
Park, playground, or sports or athletic area	46 (1.1)	<0.1	262 (1.6)	0.2	**308 (1.5)**	**0.1**
Hotel or motel	71 (1.7)	<0.1	177 (1.1)	0.1	**248 (1.2)**	**<0.1**
Jail or prison	1 (<1.0)	—	184 (1.1)	0.1	**185 (<1.0)**	**<0.1**
Abandoned house, building, or warehouse	17 (<1.0)	—	84 (<1.0)	<0.1	**101 (<1.0)**	**<0.1**
Other location^¶¶^	142 (3.4)	—	624 (3.8)	—	**766 (3.7)**	**—**
Unknown	176 (4.3)	—	885 (5.4)	—	**1,061 (5.2)**	**—**
**Incident characteristic**						
Emergency medical services present	2,878 (69.7)	1.9	12,308 (75.0)	8.1	**15,186 (73.9)**	**5.0**
Injured at victim’s home	1,986 (48.1)	1.3	3,157 (19.2)	2.1	**5,143 (25.0)**	**1.7**
Child present or witnessed incident	661 (16.0)	0.4	1,226 (7.5)	0.8	**1,887 (9.2)**	**0.6**
Victim was suspected of alcohol use preceding the incident	295 (7.1)	0.2	1,244 (7.6)	0.8	**1,539 (7.5)**	**0.5**
Victim was experiencing homelessness	87 (2.1)	<0.1	530 (3.2)	0.4	**617 (3.0)**	**0.2**
Victim was injured at work or while working	80 (1.9)	<0.1	345 (2.1)	0.2	**425 (2.1)**	**0.1**
Victim was in public custody when injury occurred	14 (<1.0)	—	298 (1.8)	0.2	**312 (1.5)**	**0.1**
Victim was recently released from an institutional setting	55 (1.3)	<0.1	230 (1.4)	0.2	**285 (1.4)**	**<0.1**
Victim was experiencing housing instability	62 (1.5)	<0.1	212 (1.3)	0.1	**274 (1.3)**	**<0.1**
Relationship of victim to suspect***						
Spouse or intimate partner (current or former)	1,315 (53.4)	0.9	369 (6.9)	0.2	**1,684 (21.6)**	**0.6**
Acquaintance or friend	207 (8.4)	0.1	1,476 (27.6)	1.0	**1,683 (21.5)**	**0.6**
Other person known to victim, but exact relationship was unclear	191 (7.8)	0.1	1,242 (23.2)	0.8	**1,433 (18.3)**	**0.5**
Stranger	177 (7.2)	0.1	1,083 (20.2)	0.7	**1,260 (16.1)**	**0.4**
Other relative	142 (5.8)	<0.1	428 (8.0)	0.3	**570 (7.3)**	**0.2**
Child^†††^	196 (8.0)	0.1	295 (5.5)	0.2	**491 (6.3)**	**0.2**
Parent^†††^	202 (8.2)	0.1	267 (5.0)	0.2	**469 (6.0)**	**0.2**
Rival gang member	1 (<1.0)	—	78 (1.5)	<0.1	**79 (1.0)**	**<0.1**
Child of suspect's intimate partner (e.g., child killed by mother's boyfriend)	25 (1.0)	<0.1	38 (<1.0)	<0.1	**63 (<1.0)**	**<0.1**
Other relationship^§§§^	5 (<1.0)	—	75 (1.4)	—	**80 (1.0)**	**—**
**Total**	**4,130 (100)**	**2.7**	**16,410 (100)**	**10.8**	**20,540 (100)**	**6.7**

* Percentages might not total 100% because of rounding.

^†^ Per 100,000 population.

^§^ Sex was known for all decedents.

^¶^ The following sentence can be used as a guide for interpreting victim-suspect relationship: “The victim is the ____________ of the suspect.” For example, when a parent kills a child, the relationship is “child” not “parent” (“The victim is the child of the suspect”). Note that this sentence is intended to be a general guide. However, certain relationships might not be included by this sentence (e.g., other person known to victim or victim was law enforcement officer killed in the line of duty).

** Data for California are for deaths that occurred in 31 counties (Alameda, Alpine, Amador, Butte, Fresno, Humboldt, Imperial, Kings, Lake, Lassen, Los Angeles, Madera, Mendocino, Merced, Mono, Orange, Placer, Riverside, Sacramento, San Benito, San Diego, San Francisco, San Luis Obispo, San Mateo, Santa Cruz, Shasta, Solano, Sonoma, Tehama, Ventura, and Yolo), data for Florida are for deaths that occurred in 38 counties (Alachua, Bay, Brevard, Calhoun, Charlotte, Citrus, Escambia, Flagler, Gilchrist, Hardee, Hernando, Highlands, Hillsborough, Holmes, Indian River, Jackson, Lake, Levy, Manatee, Marion, Martin, Okaloosa, Okeechobee, Orange, Osceola, Palm Beach, Pinellas, Polk, Putnam, St. Johns, St. Lucie, Santa Rosa, Seminole, Sumter, Union, Volusia, Walton, and Washington), and data for Texas are for deaths that occurred in 13 counties (Bell, Bexar, Collin, Dallas, Denton, El Paso, Fort Bend, Harris, Montgomery, Nueces, Tarrant, Travis, and Williamson). Denominators for the rates for California, Florida, and Texas represent only the populations of the counties from which the data were collected.

^††^ Dashes indicate cell data are suppressed because number of decedents is <20 or characteristic response is “other” or “unknown.”

^§§^ Persons of Hispanic or Latino (Hispanic) origin might be of any race but are categorized as Hispanic; all racial groups are non-Hispanic.

^¶¶^ Other location includes (in descending order): supervised residential facility; preschool, school, college, or school bus; industrial or construction area; hospital or medical facility; office building; synagogue, church, or temple; farm; railroad tracks; bridge; cemetery, graveyard, or other burial ground; and other unspecified location.

*** Percentage is based on the number of homicide decedents with a known victim-to-suspect relationship (n = 7,812 [38.0%]; 5,351 [32.6%] males and 2,461 [59.6%] females); victim-suspect relationship was unknown for 12,728 decedents.

^†††^ Includes adoptive family members (e.g., adopted child), stepfamily members (e.g., stepparent), and foster family members (e.g., foster child).

^§§§^ Other victim-suspect relationship includes (in descending order): intimate partner of suspect's parent (e.g., teenager kills his mother’s boyfriend) and victim was a law enforcement officer injured in the line of duty.

The homicide rates were higher among males than females across all age groups, and the rate was highest among adults aged 20–24 years (14.1 per 100,000 population) (Table 4). The homicide rate for males was four times the rate for females (10.8 versus 2.7 per 100,000 population). Among males, the rate of homicide was highest among adults aged 20–24 years (23.4), followed by 25–29 years and 30–34 years (both 20.7). Among females, the rate of homicide was highest among infants (aged <1 year; 5.7). Among all infants and children aged <10 years who were homicide victims, the homicide rate for infants (6.8) was 3.6 times the rate for children aged 1–4 years (1.9) and 7.6 times the rate for children aged 5–9 years (0.9).

Black persons accounted for 58.2% of male homicide victims and 39.8% of female homicide victims (Table 4). Black males had the highest rate of homicide compared with males in all other racial and ethnic groups (50.6 per 100,000 population); this rate was 28.1 times the rate for Asian males (1.8), 14.5 times the rate for White males (3.5), 4.6 times the rate for NH/PI males (11.1), 4.7 times the rate for Hispanic males (10.7), 2.6 times the rate for AI/AN males (19.7), and 9.2 times the rate for males of more than one race (5.5). Among females, the homicide rate was also highest among Black females (8.0) (Table 4), followed by AI/AN females (5.0), Hispanic females (2.4), females of more than one race (2.3), White females (1.7), and Asian females (1.2).

#### Method and Location of Injury

Firearms were used in 77.8% of homicides, making them the most commonly used weapon, followed by a sharp instrument (8.7%); a blunt instrument (3.4%); personal weapons (e.g., hands, fists, or feet; 2.9%); and hanging, strangulation, or suffocation (1.8%) (Table 4). The method of injury was unknown in 2.5% of homicides. A firearm was the most common method of fatal injury for both males (81.0%) and females (65.1%); however, the firearm homicide rate for males (8.8 per 100,000 population) was 5.2 times the rate for females (1.7). For males, the next most common methods of injury apart from firearms were sharp instruments (7.8%), blunt instruments (2.9%), and personal weapons (2.6%). For females, sharp instruments (12.2%), blunt instruments (5.2%), and hanging, strangulation, or suffocation (5.1%) were additional common methods of injury. Among all homicide victims, a house or apartment was the most common location of homicide (43.4%), followed by a street or highway (20.1%), a motor vehicle (9.9%), a commercial or retail area (5.7%), and a parking lot, public garage, or public transport (4.4%). A larger proportion of homicides of females (63.6%) than among males (38.3%) occurred at a house or apartment, whereas a larger proportion of homicides of males (23.1%) than females (8.4%) occurred on a street or highway.

#### Incident Characteristics

Emergency medical services responded to the scene for a large percentage of homicide victims (73.9%) (Table 4). A larger proportion of homicides of females than males occurred at the victim’s home (48.1% and 19.2%, respectively) and involved a child who was present or witnessed the incident (16.0% and 7.5%, respectively). Among all homicide victims, 7.5% of victims were suspected of alcohol use preceding the incident. A small proportion of all homicide victims were experiencing homelessness (3.0%), were at work or fatally injured while working (2.1%), or were in public custody at the time they were fatally injured (1.5%).

#### Victim-Suspect Relationship

The relationship of the victim to the suspect was known for 38.0% of homicides (32.6% of males and 59.6% of females) (Table 4). For males, when the relationship was known, the victim-suspect relationship most often was an acquaintance or friend (27.6%); another person known to the victim, but the exact nature of the relationship was unclear (23.2%); a stranger (20.2%); a relative other than a parent or child (8.0%); or a current or former intimate partner (6.9%). For females, when the relationship was known, approximately half (53.4%) of suspects were a current or former intimate partner, followed by an acquaintance or friend (8.4%); a parent (8.2%); a child (8.0%); another person known to victim, but the exact nature of the relationship was unclear (7.8%); or a stranger (7.2%).

#### Precipitating Circumstances

Precipitating circumstances were identified in 73.7% of homicides (Table 5). More than one third of homicides with known circumstances were precipitated by an argument or conflict (39.4%), and 16.5% of homicides with known circumstances were related to intimate partner violence (IPV). IPV-related deaths include deaths related to conflict or violence between current or former intimate partners and also include deaths associated with IPV that are not deaths of the intimate partners themselves (e.g., a former boyfriend killing an ex-partner’s new boyfriend). Homicides also were commonly precipitated by another crime (21.7%); in 60.2% of those cases, the crime was in progress at the time of the incident. The most frequent types of precipitating crimes were assault or another homicide (43.7%), robbery (28.5%), drug trade (i.e., the buying, selling, or passing of drugs from one person to another in exchange for goods or money; 11.5%), burglary (10.8%), motor vehicle theft (5.8%), rape or sexual assault (2.6%), and arson (1.0%) (Supplementary Table 5). A victim who was known to authorities (15.6%), a physical fight between two persons (14.8%), relationship problems other than intimate partner relationships (14.5%), and a drive-by shooting (11.0%) were other common precipitating circumstances (Table 5). A recent or impending crisis during the previous or upcoming 2 weeks was present for 8.4% of decedents (Table 5) (Supplementary Table 6). Toxicology results in homicide deaths are available (Supplementary Table 7).

**TABLE 5:** Number* and percentage^†^ of homicides, by decedent sex and precipitating circumstances — National Violent Death Reporting System, 50 states^§^ and District of Columbia, 2023

Precipitating circumstance	Female	Male	Total
	No. (%)	No. (%)	No. (%)
**Mental health and substance use**			
Substance use problem (excludes alcohol)	302 (9.2)	1,408 (11.9)	**1,710 (11.3)**
Current diagnosed mental health problem	343 (10.5)	735 (6.2)	**1,078 (7.1)**
Alcohol problem	113 (3.5)	487 (4.1)	**600 (4.0)**
History of ever being treated for a mental health or substance use problem	154 (4.7)	346 (2.9)	**500 (3.3)**
Current mental health or substance use treatment	95 (2.9)	188 (1.6)	**283 (1.9)**
Current depressed mood	24 (<1.0)	37 (<1.0)	**61 (<1.0)**
Nonadherence to mental health or substance use treatment	11 (<1.0)	48 (<1.0)	**59 (<1.0)**
Other addiction (e.g., gambling or sex)	2 (<1.0)	9 (<1.0)	**11 (<1.0)**
**Interpersonal factor**			
Intimate partner violence related	1,520 (46.5)	971 (8.2)	**2,491 (16.5)**
Other relationship problem (nonintimate and nonfamily)	156 (4.8)	1,092 (9.2)	**1,248 (8.2)**
Family relationship problem	299 (9.1)	650 (5.5)	**949 (6.3)**
Jealousy (lovers’ triangle)	117 (3.6)	239 (2.0)	**356 (2.4)**
Victim of interpersonal violence during past month^¶^	140 (4.3)	94 (<1.0)	**234 (1.5)**
Perpetrator of interpersonal violence during past month^¶^	18 (<1.0)	151 (1.3)	**169 (1.1)**
**Life stressor**			
Argument or conflict	1,131 (34.6)	4,830 (40.7)	**5,961 (39.4)**
Victim known to authorities	395 (12.1)	1,973 (16.6)	**2,368 (15.6)**
Physical fight (two persons, not a brawl)	299 (9.1)	1,941 (16.4)	**2,240 (14.8)**
Crisis during previous or upcoming 2 weeks	326 (10.0)	949 (8.0)	**1,275 (8.4)**
Household had contact with local authorities	304 (9.3)	317 (2.7)	**621 (4.1)**
Family stressor	71 (2.2)	102 (<1.0)	**173 (1.1)**
History of child abuse or neglect	60 (1.8)	100 (<1.0)	**160 (1.1)**
Exposure to disaster	5 (<1.0)	30 (<1.0)	**35 (<1.0)**
Living transition or loss of independent living	8 (<1.0)	4 (<1.0)	**12 (<1.0)**
**Crime and criminal activity**			
Precipitated by another crime	493 (15.1)	2,792 (23.5)	**3,285 (21.7)**
Crime in progress**	286 (58.0)	1,693 (60.6)	**1,979 (60.2)**
Drug involvement	111 (3.4)	1,049 (8.8)	**1,160 (7.7)**
Gang related	61 (1.9)	716 (6.0)	**777 (5.1)**
**Homicide event**			
Drive-by shooting	211 (6.5)	1,458 (12.3)	**1,669 (11.0)**
Victim used a weapon	68 (2.1)	1,255 (10.6)	**1,323 (8.7)**
Walk-by assault	88 (2.7)	821 (6.9)	**909 (6.0)**
Caregiver abuse or neglect that led to death	379 (11.6)	391 (3.3)	**770 (5.1)**
Justifiable self-defense	20 (<1.0)	505 (4.3)	**525 (3.5)**
Suspect with mental illness**^††^**	208 (6.6)	266 (2.6)	**474 (3.5)**
Brawl	30 (<1.0)	319 (2.7)	**349 (2.3)**
Victim was a bystander	120 (3.7)	184 (1.6)	**304 (2.0)**
Random violence	63 (1.9)	222 (1.9)	**285 (1.9)**
Victim was an intervener assisting a crime victim	18 (<1.0)	153 (1.3)	**171 (1.1)**
Stalking	27 (<1.0)	31 (<1.0)	**58 (<1.0)**
Prostitution	27 (<1.0)	29 (<1.0)	**56 (<1.0)**
Victim was a police officer on duty	5 (<1.0)	38 (<1.0)	**43 (<1.0)**
Mercy killing	13 (<1.0)	6 (<1.0)	**19 (<1.0)**
Hate crime	4 (<1.0)	15 (<1.0)	**19 (<1.0)**
Terrorist attack	0 (—)	0 (—)	**0 (—)**
**Child victim incident^§§^**			
Previous Child Protective Services report on victim's household	47 (11.7)	51 (4.5)	**98 (6.4)**
Substance use in victim's household	25 (6.2)	42 (3.7)	**67 (4.4)**
Corporal punishment	4 (<1.0)	10 (<1.0)	**14 (<1.0)**
**Total^¶¶^**	**3,271 (79.2)**	**11,866 (72.3)**	**15,137 (73.7)**

* Includes homicides with one or more precipitating circumstances. Total numbers do not equal the sums of the columns because more than one circumstance could have been present per decedent.

^†^ Denominator includes those homicides with one or more precipitating circumstances. The sums of percentages in columns exceed 100% because more than one circumstance could have been present per decedent.

^§^ Data for California are for deaths that occurred in 31 counties (Alameda, Alpine, Amador, Butte, Fresno, Humboldt, Imperial, Kings, Lake, Lassen, Los Angeles, Madera, Mendocino, Merced, Mono, Orange, Placer, Riverside, Sacramento, San Benito, San Diego, San Francisco, San Luis Obispo, San Mateo, Santa Cruz, Shasta, Solano, Sonoma, Tehama, Ventura, and Yolo), data for Florida are for deaths that occurred in 38 counties (Alachua, Bay, Brevard, Calhoun, Charlotte, Citrus, Escambia, Flagler, Gilchrist, Hardee, Hernando, Highlands, Hillsborough, Holmes, Indian River, Jackson, Lake, Levy, Manatee, Marion, Martin, Okaloosa, Okeechobee, Orange, Osceola, Palm Beach, Pinellas, Polk, Putnam, St. Johns, St. Lucie, Santa Rosa, Seminole, Sumter, Union, Volusia, Walton, and Washington), and data for Texas are for deaths that occurred in 13 counties (Bell, Bexar, Collin, Dallas, Denton, El Paso, Fort Bend, Harris, Montgomery, Nueces, Tarrant, Travis, and Williamson).

^¶^ Interpersonal violence includes but is not limited to intimate partner violence.

** Denominator includes those decedents involved in an incident that was precipitated by another crime.

^††^ Denominator includes those decedents with informative (not missing or unknown) data in one or more of these suspect variables: sex, age, race and ethnicity, or victim-suspect relationship: 13,566 total (10,410 males and 3,156 females).

^§§^ Circumstance variables in this category are applicable exclusively to children. Denominator includes decedents aged <18 years for whom circumstances are known (1,130 males, 401 females, and 1,531 total). Circumstances were unknown for 524 decedents aged <18 years (443 males and 81 females).

^¶¶^ Circumstances were unknown for 5,403 decedents (4,544 males and 859 females); total number of homicide decedents = 20,540 (16,410 males and 4,130 females).

Among the identified homicide circumstances, multiple differences were noted by the decedents’ sex, with IPV accounting for the largest percentage-point difference. When the victim-suspect relationship was known, the suspect was a current or former intimate partner in 53.4% of homicides of females and 6.9% of homicides of males (data not shown). When all homicides in which IPV was a contributing circumstance are included, either directed at the intimate partner themselves or corollary victims, approximately 46.5% of homicides of females and 8.2% of homicides of males were IPV-related (Table 5). Females were more often the direct victims of IPV-related homicides, whereas males were more often corollary victims. A larger proportion of homicides of females than males also resulted from caregiver abuse or neglect (11.6% versus 3.3%) or involved a household that had contact with local authorities (9.3% versus 2.7%) (Table 5). A larger proportion of homicides of males than females were preceded by a physical fight (16.4% versus 9.1%), involved drugs (8.8% versus 3.4%), or were related to gang activity (6.0% versus 1.9%). A larger proportion of male homicide victims (10.6%) than female homicide victims (2.1%) also were reported to have used a weapon during the incident.

Known circumstances of incidents were identified in 1,531 (74.5%) homicides of children aged <18 years (Table 5). With respect to two circumstances that only applied to child decedents, a larger proportion of female victims’ households had previous Child Protective Services reports (11.7% versus 4.5%) or reported substance use problems in their households (6.2% versus 3.7%) compared with male victims’ households.

### Legal Intervention Deaths

#### Demographics

For 2023, NVDRS collected data on 1,073 incidents involving 1,083 legal intervention deaths (Supplementary Table 2). Nearly all legal intervention deaths were among males (95.6%) (Table 6). The highest rate of legal intervention death by age group was among men aged 30–34 years (1.6 per 100,000 population), followed by men aged 35–44 years (1.4) and 25–29 years (1.1). Although White males accounted for 48.1% of all male legal intervention deaths, AI/AN males had the highest legal intervention death rate (2.1), representing a rate over 4.2 times that of White males (0.5). The legal intervention death rate for Black males (1.4) was 2.8 times the rate for White males. The legal intervention death rate for Hispanic males was 0.8 per 100,000 population.

**TABLE 6:** Number, percentage,* and rate^†^ of legal intervention^§^ deaths, by selected demographics of decedent, method of injury, location of injury, and incident characteristics — National Violent Death Reporting System, 50 states^¶^ and District of Columbia, 2023

Characteristic	Female		Male		Total	
	No. (%)	Rate	No. (%)	Rate	No. (%)	Rate
**Age group, yrs**						
<10	0 (—)	—**	0 (—)	—	**0 (—)**	**—**
10–14	0 (—)	—	1 (<1.0)	—	**1 (<1.0)**	**—**
15–19	2 (4.2)	—	59 (5.7)	0.6	**61 (5.6)**	**0.3**
20–24	7 (14.6)	—	99 (9.6)	1.0	**106 (9.8)**	**0.5**
25–29	7 (14.6)	—	118 (11.4)	1.1	**125 (11.5)**	**0.6**
30–34	5 (10.4)	—	178 (17.2)	1.6	**183 (16.9)**	**0.8**
35–44	13 (27.1)	—	285 (27.5)	1.4	**298 (27.5)**	**0.7**
45–54	9 (18.8)	—	168 (16.2)	0.9	**177 (16.3)**	**0.5**
55–64	3 (6.3)	—	78 (7.5)	0.4	**81 (7.5)**	**0.2**
65–74	2 (4.2)	—	33 (3.2)	0.2	**35 (3.2)**	**0.1**
75–84	0 (—)	—	15 (1.4)	—	**15 (1.4)**	**—**
≥85	0 (—)	—	1 (<1.0)	—	**1 (<1.0)**	**—**
**Race and ethnicity^††^**						
American Indian or Alaska Native	1 (2.1)	—	24 (2.3)	2.1	**25 (2.3)**	**1.1**
Asian	0 (—)	—	12 (1.2)	—	**12 (1.1)**	**—**
Black or African American	11 (22.9)	—	268 (25.9)	1.4	**279 (25.8)**	**0.7**
Hispanic or Latino	8 (16.7)	—	209 (20.2)	0.8	**217 (20.0)**	**0.4**
Native Hawaiian or Pacific Islander	0 (—)	—	3 (<1.0)	—	**3 (<1.0)**	**—**
White	26 (54.2)	<0.1	498 (48.1)	0.5	**524 (48.4)**	**0.3**
More than one race	1 (2.1)	—	14 (1.4)	—	**15 (1.4)**	**—**
Unspecified or unknown	1 (2.1)	—	7 (<1.0)	—	**8 (<1.0)**	**—**
**Method of injury**						
Firearm	39 (81.3)	<0.1	894 (86.4)	0.6	**933 (86.1)**	**0.3**
Motor vehicle (e.g., bus, motorcycle, or other transport vehicle)	8 (16.7)	—	65 (6.3)	<0.1	**73 (6.7)**	**<0.1**
Poisoning	0 (—)	—	13 (1.3)	—	**13 (1.2)**	**—**
Personal weapon (e.g., hands, feet, or fists)	0 (—)	—	8 (<1.0)	—	**8 (<1.0)**	**—**
Drowning	0 (—)	—	8 (<1.0)	—	**8 (<1.0)**	**—**
Blunt instrument	0 (—)	—	6 (<1.0)	—	**6 (<1.0)**	**—**
Sharp instrument	0 (—)	—	2 (<1.0)	—	**2 (<1.0)**	**—**
Hanging, strangulation, or suffocation	0 (—)	—	2 (<1.0)	—	**2 (<1.0)**	**—**
Fall	0 (—)	—	2 (<1.0)	—	**2 (<1.0)**	**—**
Fire or burns	0 (—)	—	1 (<1.0)	—	**1 (<1.0)**	**—**
Other (e.g., Taser, electrocution, or nail gun)	0 (—)	—	11 (1.1)	—	**11 (1.0)**	**—**
Unknown	1 (2.1)	—	23 (2.2)	—	**24 (2.2)**	**—**
**Location of injury**						
House or apartment	16 (33.3)	—	407 (39.3)	0.3	**423 (39.1)**	**0.1**
Street or highway	9 (18.8)	—	255 (24.6)	0.2	**264 (24.4)**	**<0.1**
Motor vehicle	14 (29.2)	—	111 (10.7)	<0.1	**125 (11.5)**	**<0.1**
Parking lot, public garage, or public transport	1 (2.1)	—	47 (4.5)	<0.1	**48 (4.4)**	**<0.1**
Commercial or retail area	2 (4.2)	—	45 (4.3)	<0.1	**47 (4.3)**	**<0.1**
Natural area	1 (2.1)	—	41 (4.0)	<0.1	**42 (3.9)**	**<0.1**
Other location^§§^	4 (8.3)	—	86 (8.3)	—	**90 (8.3)**	**—**
Unknown	1 (2.1)	—	43 (4.2)	—	**44 (4.1)**	**—**
**Incident characteristic**						
Emergency medical services present	45 (93.8)	<0.1	874 (84.4)	0.6	**919 (84.9)**	**0.3**
Victim was in public custody when injury occurred	12 (25.0)	—	339 (32.8)	0.2	**351 (32.4)**	**0.1**
Injured at victim’s home	14 (29.2)	—	250 (24.2)	0.2	**264 (24.4)**	**<0.1**
Victim was suspected of alcohol use preceding the incident	7 (14.6)	—	123 (11.9)	<0.1	**130 (12.0)**	**<0.1**
Child present or witnessed incident	6 (12.5)	—	65 (6.3)	<0.1	**71 (6.6)**	**<0.1**
Victim was recently released from an institutional setting	1 (2.1)	—	52 (5.0)	<0.1	**53 (4.9)**	**<0.1**
Victim was experiencing homelessness	1 (2.1)	—	39 (3.8)	<0.1	**40 (3.7)**	**<0.1**
Victim was experiencing housing instability	2 (4.2)	—	25 (2.4)	<0.1	**27 (2.5)**	**<0.1**
Victim was injured at work or while working	0 (—)	—	5 (<1.0)	—	**5 (<1.0)**	**—**
**Total**	**48 (100)**	**<0.1**	**1,035 (100)**	**0.7**	**1,083 (100)**	**0.4**

* Percentages might not total 100% because of rounding.

^†^ Per 100,000 population.

^§^ The term “legal intervention” does not denote the lawfulness or legality of the circumstances surrounding the death.

^¶^ Data for California are for deaths that occurred in 31 counties (Alameda, Alpine, Amador, Butte, Fresno, Humboldt, Imperial, Kings, Lake, Lassen, Los Angeles, Madera, Mendocino, Merced, Mono, Orange, Placer, Riverside, Sacramento, San Benito, San Diego, San Francisco, San Luis Obispo, San Mateo, Santa Cruz, Shasta, Solano, Sonoma, Tehama, Ventura, and Yolo), data for Florida are for deaths that occurred in 38 counties (Alachua, Bay, Brevard, Calhoun, Charlotte, Citrus, Escambia, Flagler, Gilchrist, Hardee, Hernando, Highlands, Hillsborough, Holmes, Indian River, Jackson, Lake, Levy, Manatee, Marion, Martin, Okaloosa, Okeechobee, Orange, Osceola, Palm Beach, Pinellas, Polk, Putnam, St. Johns, St. Lucie, Santa Rosa, Seminole, Sumter, Union, Volusia, Walton, and Washington), and data for Texas are for deaths that occurred in 13 counties (Bell, Bexar, Collin, Dallas, Denton, El Paso, Fort Bend, Harris, Montgomery, Nueces, Tarrant, Travis, and Williamson). Denominators for the rates for California, Florida, and Texas represent only the populations of the counties from which the data were collected.

** Dashes indicate cell data are suppressed because number of decedents is <20 or characteristic response is “other” or “unknown.”

^††^ Persons of Hispanic or Latino (Hispanic) origin might be of any race but are categorized as Hispanic; all racial groups are non-Hispanic.

^§§^ Other location includes (in descending order): hotel or motel; bar or nightclub; hospital or medical facility; jail or prison; industrial or construction area; preschool, school, college, or school bus; supervised residential facility; railroad tracks; office building; abandoned house, building, or warehouse; park, playground, or sports or athletic area; farm; bridge; and other unspecified location.

#### Method and Location of Injury

A firearm was used in the majority (86.1%) of legal intervention deaths (Table 6). Legal intervention deaths occurred most frequently in a house or apartment (39.1%), followed by a street or highway (24.4%) or a motor vehicle (11.5%).

#### Incident Characteristics

Among all legal intervention deaths, emergency medical services responded to the scene for a large percentage of deaths (84.9%), and approximately one third of decedents were in public custody when the injury occurred (32.4%) (Table 6). Approximately one fourth of legal intervention deaths occurred at the decedent’s own home (24.4%); 12.0% of decedents were suspected of alcohol use preceding the incident, and 6.6% of deaths had a child present or witnessing the incident. A small proportion of legal intervention deaths involved decedents experiencing homelessness (3.7%) or housing instability (2.5%) at the time of death.

#### Precipitating Circumstances

Precipitating circumstances were identified in 93.9% of legal intervention deaths (Table 7). The decedent reportedly used a weapon in 69.7% of legal intervention death cases. The decedent was known to the authorities in over one third of deaths (35.8%). In 22.2% of legal intervention deaths with known circumstances, the decedent reportedly had a substance use problem (other than alcohol), 20.8% of decedents reportedly had a current diagnosed mental health problem, 12.1% had a history of mental health or substance use treatment, and 9.2% had an alcohol problem. An argument or conflict or physical fight precipitated 16.9% and 6.1% of legal intervention deaths, respectively. A recent or impending crisis during the previous or upcoming 2 weeks was reported in 11.0% of legal intervention deaths (Table 7) (Supplementary Table 8). Among legal intervention deaths with known circumstances, being a perpetrator of interpersonal violence (including but not limited to IPV) during the past month (9.1%) was another notable precipitating circumstance, as were being related to IPV specifically (7.3%), family relationship problems (5.6%), and drug activity (4.1%) (Table 7). Toxicology results for legal intervention deaths are available (Supplementary Table 9).

**TABLE 7:** Number* and percentage^†^ of legal intervention^§^ deaths, by decedent sex and precipitating circumstances — National Violent Death Reporting System, 50 states^¶^ and District of Columbia, 2023

Precipitating circumstance	Female	Male	Total
	No. (%)	No. (%)	No. (%)
**Mental health and substance use**			
Substance use problem (excludes alcohol)	6 (13.6)	220 (22.6)	**226 (22.2)**
Current diagnosed mental health problem	12 (27.3)	200 (20.6)	**212 (20.8)**
History of ever being treated for a mental health or substance use problem	8 (18.2)	115 (11.8)	**123 (12.1)**
Alcohol problem	3 (6.8)	91 (9.4)	**94 (9.2)**
Current mental health or substance use treatment	5 (11.4)	73 (7.5)	**78 (7.7)**
Current depressed mood	3 (6.8)	27 (2.8)	**30 (2.9)**
Nonadherence to mental health or substance use treatment	1 (2.3)	26 (2.7)	**27 (2.7)**
Other addiction (e.g., gambling or sex)	0 (—)	2 (<1.0)	**2 (<1.0)**
**Interpersonal factor**			
Perpetrator of interpersonal violence during past month**	4 (9.1)	89 (9.1)	**93 (9.1)**
Intimate partner violence related	4 (9.1)	70 (7.2)	**74 (7.3)**
Family relationship problem	3 (6.8)	54 (5.5)	**57 (5.6)**
Other relationship problem (nonintimate and nonfamily)	2 (4.5)	27 (2.8)	**29 (2.9)**
Victim of interpersonal violence during past month**	0 (—)	1 (<1.0)	**1 (<1.0)**
Jealousy (lovers’ triangle)	0 (—)	1 (<1.0)	**1 (<1.0)**
**Life stressor**			
Victim known to authorities	15 (34.1)	349 (35.9)	**364 (35.8)**
Argument or conflict	9 (20.5)	163 (16.8)	**172 (16.9)**
Crisis during previous or upcoming 2 weeks	5 (11.4)	107 (11.0)	**112 (11.0)**
Physical fight (two persons, not a brawl)	5 (11.4)	57 (5.9)	**62 (6.1)**
Household had contact with local authorities	3 (6.8)	38 (3.9)	**41 (4.0)**
Family stressor	1 (2.3)	10 (1.0)	**11 (1.1)**
History of child abuse or neglect	0 (—)	5 (<1.0)	**5 (<1.0)**
Exposure to disaster	0 (—)	4 (<1.0)	**4 (<1.0)**
**Crime and criminal activity**			
Drug involvement	0 (—)	42 (4.3)	**42 (4.1)**
Gang related	1 (2.3)	11 (1.1)	**12 (1.2)**
**Legal intervention event**			
Victim used a weapon	29 (65.9)	680 (69.9)	**709 (69.7)**
Brawl	0 (—)	7 (<1.0)	**7 (<1.0)**
Stalking	0 (—)	4 (<1.0)	**4 (<1.0)**
Victim was a bystander	0 (—)	2 (<1.0)	**2 (<1.0)**
Victim was an intervener assisting a crime victim	0 (—)	2 (<1.0)	**2 (<1.0)**
Caretaker abuse or neglect	0 (—)	2 (<1.0)	**2 (<1.0)**
Random violence	0 (—)	1 (<1.0)	**1 (<1.0)**
**Total^††^**	**44 (91.7)**	**973 (94.0)**	**1,017 (93.9)**

* Includes deaths with one or more precipitating circumstances. Total numbers do not equal the sums of the columns because more than one circumstance could have been present per decedent.

^†^ Denominator includes those deaths with one or more precipitating circumstances. The sums of percentages in columns exceed 100% because more than one circumstance could have been present per decedent.

^§^ The term “legal intervention” does not denote the lawfulness or legality of the circumstances surrounding the death.

^¶^ Data for California are for deaths that occurred in 31 counties (Alameda, Alpine, Amador, Butte, Fresno, Humboldt, Imperial, Kings, Lake, Lassen, Los Angeles, Madera, Mendocino, Merced, Mono, Orange, Placer, Riverside, Sacramento, San Benito, San Diego, San Francisco, San Luis Obispo, San Mateo, Santa Cruz, Shasta, Solano, Sonoma, Tehama, Ventura, and Yolo), data for Florida are for deaths that occurred in 38 counties (Alachua, Bay, Brevard, Calhoun, Charlotte, Citrus, Escambia, Flagler, Gilchrist, Hardee, Hernando, Highlands, Hillsborough, Holmes, Indian River, Jackson, Lake, Levy, Manatee, Marion, Martin, Okaloosa, Okeechobee, Orange, Osceola, Palm Beach, Pinellas, Polk, Putnam, St. Johns, St. Lucie, Santa Rosa, Seminole, Sumter, Union, Volusia, Walton, and Washington), and data for Texas are for deaths that occurred in 13 counties (Bell, Bexar, Collin, Dallas, Denton, El Paso, Fort Bend, Harris, Montgomery, Nueces, Tarrant, Travis, and Williamson).

** Interpersonal violence includes but is not limited to intimate partner violence.

^††^ Circumstances were unknown for 66 decedents (62 males and four females); total number of legal intervention deaths = 1,083 (1,035 males and 48 females).

### Unintentional Firearm Injury Deaths

#### Demographics

In 2023, NVDRS collected data on 542 incidents involving 542 unintentional firearm injury deaths (Supplementary Table 2). Nearly half (n = 253 [46.7%]) of these deaths were self-inflicted, and 191 deaths (35.2%) were inflicted by another person; for the remaining 98 deaths (18.0%), whether the injury was self-inflicted or inflicted by another person was unknown (data not shown). Males accounted for 83.6% of decedents (Table 8). Nearly half of decedents were White persons (47.4%), followed by Black persons (34.7%). Persons aged ≤24 years accounted for more than half (approximately 55%) of all unintentional firearm injury deaths and persons aged <18 years accounted for approximately 27.4%.

**TABLE 8:** Number and percentage* of unintentional firearm injury deaths, by selected demographics of decedent, location of injury, type of firearm, and incident characteristics — National Violent Death Reporting System, 50 states^†^ and District of Columbia, 2023

Characteristic	No. (%)
**Sex**	
Female	89 (16.4)
Male	453 (83.6)
**Race and ethnicity^§^**	
American Indian or Alaska Native	13 (2.4)
Asian	3 (<1.0)
Black or African American	188 (34.7)
Hispanic or Latino	69 (12.7)
Native Hawaiian or Pacific Islander	1 (<1.0)
White	257 (47.4)
More than one race	10 (1.8)
Unspecified or unknown	1 (<1.0)
**Age group, yrs**	
<1	1 (<1.0)
1–4	52 (9.6)
5–9	26 (4.8)
10–14	47 (8.7)
15–19	104 (19.2)
20–24	68 (12.5)
25–29	46 (8.5)
30–34	36 (6.6)
35–44	45 (8.3)
45–54	40 (7.4)
55–64	33 (6.1)
65–74	21 (3.9)
75–84	20 (3.7)
≥85	3 (<1.0)
**Location of injury**	
House or apartment	393 (72.5)
Motor vehicle	43 (7.9)
Natural area	23 (4.2)
Street or highway	19 (3.5)
Commercial or retail area	7 (1.3)
Hotel or motel	7 (1.3)
Park, playground, or sports or athletic area	6 (1.1)
Parking lot, public garage, or public transport	4 (<1.0)
Farm	4 (<1.0)
Industrial or construction area	1 (<1.0)
Preschool, school, college, or school bus	1 (<1.0)
Other unspecified location	9 (1.7)
Unknown	25 (4.6)
**Firearm type**	
Handgun	336 (62.0)
Rifle	41 (7.6)
Shotgun	31 (5.7)
Other	2 (<1.0)
Unknown	132 (24.4)
**Incident characteristic**	
Emergency medical services present	399 (73.6)
Injured at victim’s home	268 (49.4)
Child present or witnessed incident	130 (24.0)
Victim was suspected of alcohol use preceding the incident	83 (15.3)
Victim was injured at work or while working	8 (1.5)
Victim was recently released from an institutional setting	6 (1.1)
Victim was experiencing homelessness	3 (<1.0)
Victim was in public custody when injury occurred	3 (<1.0)
**Total**	**542 (100)**

* Percentages might not total 100% because of rounding.

^†^ Data for California are for deaths that occurred in 31 counties (Alameda, Alpine, Amador, Butte, Fresno, Humboldt, Imperial, Kings, Lake, Lassen, Los Angeles, Madera, Mendocino, Merced, Mono, Orange, Placer, Riverside, Sacramento, San Benito, San Diego, San Francisco, San Luis Obispo, San Mateo, Santa Cruz, Shasta, Solano, Sonoma, Tehama, Ventura, and Yolo), data for Florida are for deaths that occurred in 38 counties (Alachua, Bay, Brevard, Calhoun, Charlotte, Citrus, Escambia, Flagler, Gilchrist, Hardee, Hernando, Highlands, Hillsborough, Holmes, Indian River, Jackson, Lake, Levy, Manatee, Marion, Martin, Okaloosa, Okeechobee, Orange, Osceola, Palm Beach, Pinellas, Polk, Putnam, St. Johns, St. Lucie, Santa Rosa, Seminole, Sumter, Union, Volusia, Walton, and Washington), and data for Texas are for deaths that occurred in 13 counties (Bell, Bexar, Collin, Dallas, Denton, El Paso, Fort Bend, Harris, Montgomery, Nueces, Tarrant, Travis, and Williamson).

^§^ Persons of Hispanic or Latino (Hispanic) origin might be of any race but are categorized as Hispanic; all racial groups are non-Hispanic.

#### Location of Injury and Firearm Type

Among unintentional firearm injury deaths, 72.5% occurred in a house or apartment, followed by a motor vehicle (7.9%) or a natural area (4.2%) (Table 8). The majority of unintentional firearm injury deaths involved a handgun (62.0%), followed by a rifle (7.6%) or a shotgun (5.7%). The firearm type was unknown in approximately one fourth (24.4%) of unintentional firearm injury deaths.

#### Incident Characteristics

Emergency medical services responded to the scene in approximately three fourths (73.6%) of unintentional firearm injury deaths (Table 8). Approximately one half of all unintentional firearm injury deaths occurred at the decedent’s own home (49.4%), and a child was present or witnessed the incident in 24.0% of unintentional firearm injury deaths. Decedents were suspected of alcohol use preceding the incident in 15.3% of cases.

#### Context and Circumstances of Injury

The context and circumstances of injury were identified in 84.3% of unintentional firearm injury deaths (Table 9). Among those with context and circumstance information, the context of injury was most frequently playing with a firearm (39.8%). Other contexts of injury were showing the firearm to others (14.0%), cleaning the firearm (7.9%), hunting (4.6%), and loading or unloading the firearm (3.7%). Approximately one fourth (24.3%) of unintentional firearm injury deaths were precipitated by a person unintentionally pulling the trigger; 18.8% resulted from a person mistakenly thinking the firearm was unloaded (with 8.3% occurring as a result of a disengaged magazine and 10.5% for other reasons); 3.9% of deaths were because the firearm was dropped; and 3.9% of deaths were because the firearm was mistaken for a toy.

**TABLE 9:** Number and percentage* of unintentional firearm injury deaths, by context and circumstances of injury — National Violent Death Reporting System, 50 states^†^ and District of Columbia, 2023

Characteristic	No. (%)
**Context of injury**	
Playing with firearm	182 (39.8)
Showing firearm to others	64 (14.0)
Cleaning firearm	36 (7.9)
Hunting	21 (4.6)
Loading or unloading firearm	17 (3.7)
Target shooting	4 (<1.0)
Celebratory firing	3 (<1.0)
Self-defensive shooting	1 (<1.0)
Other context of injury	84 (18.4)
**Circumstances of injury**	
Unintentionally pulled trigger	111 (24.3)
Thought firearm was unloaded (not because magazine disengaged)	48 (10.5)
Thought firearm was unloaded, magazine disengaged	38 (8.3)
Firearm was dropped	18 (3.9)
Firearm was mistaken for a toy	18 (3.9)
Firearm fired while holstering	12 (2.6)
Thought firearm safety was engaged	11 (2.4)
Firearm fired due to defect or malfunction	7 (1.5)
Bullet ricocheted	4 (<1.0)
Firearm fired while handling safety lock	3 (<1.0)
Other mechanism of injury	77 (16.8)
**Child victim incident^§^**	
Substance use in victim's household	4 (2.4)
Previous Child Protective Services report on victim's household	3 (1.8)
**Total^¶^**	**457 (84.3)**

* Percentages might exceed 100% because one or more circumstances could have been known per death. Number and percentage are reported when the number of deaths is <5 because no particular circumstance identifies a single death. Denominator includes those deaths with one or more precipitating circumstances.

^†^ Data for California are for deaths that occurred in 31 counties (Alameda, Alpine, Amador, Butte, Fresno, Humboldt, Imperial, Kings, Lake, Lassen, Los Angeles, Madera, Mendocino, Merced, Mono, Orange, Placer, Riverside, Sacramento, San Benito, San Diego, San Francisco, San Luis Obispo, San Mateo, Santa Cruz, Shasta, Solano, Sonoma, Tehama, Ventura, and Yolo), data for Florida are for deaths that occurred in 38 counties (Alachua, Bay, Brevard, Calhoun, Charlotte, Citrus, Escambia, Flagler, Gilchrist, Hardee, Hernando, Highlands, Hillsborough, Holmes, Indian River, Jackson, Lake, Levy, Manatee, Marion, Martin, Okaloosa, Okeechobee, Orange, Osceola, Palm Beach, Pinellas, Polk, Putnam, St. Johns, St. Lucie, Santa Rosa, Seminole, Sumter, Union, Volusia, Walton, and Washington), and data for Texas are for deaths that occurred in 13 counties (Bell, Bexar, Collin, Dallas, Denton, El Paso, Fort Bend, Harris, Montgomery, Nueces, Tarrant, Travis, and Williamson).

^§^ Variables in this category apply exclusively to child victims for whom circumstances were known. Denominator includes decedents aged <18 years, n = 168; circumstances were unknown for 20 child decedents.

^¶^ Circumstances were unknown for 85 decedents; total number of unintentional firearm injury decedents = 542.

Known circumstances of child victim incidents were identified in 148 (88.1%) unintentional firearm injury deaths of those aged <18 years. Substance use problems and previous Child Protective Services reports were reported in 2.4% and 1.8% of child victims’ households, respectively.

### Deaths of Undetermined Intent

#### Demographics

In 2023, NVDRS collected data on 4,920 incidents involving 4,962 deaths of undetermined intent (Supplementary Table 2). The overall rate of death of undetermined intent was 1.6 per 100,000 population. The rate of death of undetermined intent was twice as high among males (2.2) versus females (1.1) (Supplementary Table 10). Approximately two thirds (67.7%) of deaths of undetermined intent were among adults aged 30–64 years. The rate of death of undetermined intent was highest among men aged 35–44 years (3.6), followed by men aged 30–34 years (3.3) and 55–64 years (3.1). The rate of death of undetermined intent among infants (aged <1 year) was 3.0 per 100,000 population. Although White persons accounted for the majority (59.0% [1.6 per 100,000 population]) of deaths of undetermined intent, AI/AN persons had the highest rate (4.0). Among both males and females, AI/AN (5.8 and 2.3, respectively) and Black (4.6 and 1.6, respectively) persons had the highest rates of deaths of undetermined intent.

#### Method and Location of Injury

Poisoning was the most common method of injury in deaths of undetermined intent (60.8%) (see Toxicology Results of Decedent and Supplementary Table 11), followed by a firearm (6.2%), a blunt instrument (5.2%), drowning (4.9%), a motor vehicle (4.0%), a fall (3.7%), fire or burns (3.2%), and hanging, strangulation, or suffocation (2.5%). Personal weapons, sharp instruments, intentional neglect, shaking, and other methods were each used as method of injury in <1.0% of undetermined intent deaths (Supplementary Table 10). Weapon type was unknown for 6.4% of undetermined intent deaths. The majority of deaths of undetermined intent occurred in a house or apartment (59.1%), followed by a natural area (6.5%), a street or highway (5.4%), a hotel or motel (3.6%), or a motor vehicle (3.6%).

#### Incident Characteristics

Among all undetermined intent deaths, emergency medical services responded to the scene for a large percentage of deaths (73.2%) (Supplementary Table 10). Approximately one half of deaths of undetermined intent occurred at the decedent’s home (48.9%). Decedents were suspected of alcohol use preceding the incident in 12.9% of cases; 7.4% of decedents had recently been released from an institutional setting, and 5.4% of the deaths had a child present or witnessing the incident. A small proportion of deaths of undetermined intent involved decedents experiencing homelessness (6.5%) or housing instability (3.0%) at the time of death.

#### Toxicology Results of Decedent

Toxicology tests for BAC were conducted for 67.3% of decedents in deaths of undetermined intent (Supplementary Table 11). Among those with positive results for alcohol (i.e., ethanol [34.2%]), 48.3% had a BAC ≥0.08 g/dL. The proportion of decedents tested for a substance varied, and among those tested, the positive results differed by substance, as follows: amphetamines (46.9% tested, of which 35.0% were positive), anticonvulsants (36.1% tested, of which 23.7% were positive), antidepressants (39.6% tested, of which 39.4% were positive), antipsychotics (33.0% tested, of which 15.4% were positive), barbiturates (38.0% tested, of which 2.4% were positive), benzodiazepines (44.8% tested, of which 28.4% were positive), cannabis (commonly referred to as marijuana; 41.4% tested, of which 33.2% were positive), cocaine (54.8% tested, of which 39.5% were positive), muscle relaxants (32.6% tested, of which 5.3% were positive), and opioids (including illicit and prescription; 68.0% tested, of which 67.3% were positive). Carbon monoxide was tested for a substantially smaller proportion of decedents (9.5%) but was identified in a large proportion of those tested (39.4%).

#### Precipitating Circumstances

Circumstances were identified in 76.9% of deaths of undetermined intent (Supplementary Table 12). Among deaths of undetermined intent with known circumstances, 37.2% of decedents had a current diagnosed mental health problem at the time of death. Among those with a diagnosed mental health problem, the most common diagnoses were depression or dysthymia (51.7%), an anxiety disorder (28.9%), and bipolar disorder (21.8%); 7.3% had depressed mood at the time of death. Substance use problems with substances other than alcohol (63.7%) and alcohol problems (24.5%) were commonly reported circumstances. Among all deaths of undetermined intent, 20.2% of decedents were receiving mental health or substance use treatment at the time of death; 28.6% of decedents had a history of ever being treated for a mental health or substance use problem. The victim was known to authorities in 18.3% of cases. A recent or impending crisis during the preceding or upcoming 2 weeks (13.0%) (Supplementary Tables 12 and 13) and physical health problems (11.3%) were other life stressors identified in deaths of undetermined intent. Among decedents, 14.5% had a history of suicidal thoughts or plans, 8.9% had a history of attempting suicide, and 6.0% had disclosed intent to die by suicide.

Circumstances were identified in 207 (76.9%) undetermined intent deaths of children aged <18 years (Supplementary Table 12). Female decedents had a higher percentage of substance use problems in their household compared with male decedents (36.0% and 25.6%, respectively). Previous Child Protective Services involvement was more frequently reported in female decedents’ households (23.3%) than in male decedents’ households (21.5%).

### Deaths in Puerto Rico

For 2023, Puerto Rico collected data on 671 incidents involving 730 deaths (data not shown). Homicide (n = 485) accounted for the largest proportion (66.4%) and highest rate (15.1 per 100,000 population) of these deaths, followed by suicide (n = 240 [32.9%; 7.5 per 100,000 population]) (Supplementary Tables 14 and 15).

### Homicides

#### Demographics

In 2023, a total of 439 homicides of males and 46 homicides of females were reported in Puerto Rico (Supplementary Table 14). The overall homicide rate for males (29.0 per 100,000 population) was 10.7 times the rate for females (2.7). Among males, the homicide rate was highest among those aged 18–29 years (69.2) and those aged 30–44 years (56.1). Most (97.3%) homicide victims were Hispanic.

#### Method and Location of Injury

A firearm was used in the majority (90.9%) of homicides (Supplementary Table 14). A firearm was the most common method used in homicides of both males (92.9%) and females (71.7%); however, the firearm homicide rate for males (27.0 per 100,000 population) was 13.5 times the rate for females (2.0). Among males, a street or highway was the most common location of homicides (51.5%), whereas a house or apartment was the most common location of homicides for females (45.7%).

#### Incident Characteristics

Emergency medical services responded to the scene for over half of homicide victims (56.1%). A larger proportion of homicides of females than males occurred at the victim’s home (41.3% versus 5.5%, respectively).

#### Victim-Suspect Relationship

The victim-suspect relationship was known for 24.3% of homicides (Supplementary Table 14). When the relationship was known, the suspect for male victims was most often an acquaintance or friend (71.0%) or a stranger (11.8%). Among females, the suspect was most often a current or former intimate partner (76.0%).

#### Toxicology Results of Decedent

Tests for BAC were conducted for 98.4% of homicide decedents (Supplementary Table 16). Among those with positive results for alcohol (i.e., ethanol [31.4%]), 55.3% had a BAC ≥0.08 g/dL. The proportion of decedents tested for a substance varied and among those tested, the positive results differed by substance, as follows: amphetamines (<1.0% tested, of which none were positive), barbiturates (3.9% tested, of which none were positive), benzodiazepines (5.2% tested, percentage positive suppressed), cannabis (commonly referred to as marijuana; 96.7% tested, of which 38.2% were positive), cocaine (99.0% tested, of which 22.7% were positive), muscle relaxants (<1.0% tested, of which none were positive), and opioids (including illicit and prescription; 98.6% tested, of which 9.8% were positive). Carbon monoxide was tested for a substantially smaller proportion of decedents (2.3%; percentage positive suppressed).

#### Precipitating Circumstances

Precipitating circumstances were identified in 94.6% of homicides (Supplementary Table 17). Among males, 39.3% involved drugs, 39.1% were known to the authorities, and nearly one third (29.0%) involved drive-by shootings. IPV was identified as a contributing factor in 7.0% of homicides overall; IPV precipitated 52.4% of homicides of females, compared with 2.4% of homicides of males.

### Suicides

#### Demographics

In 2023, a total of 240 suicides (197 suicides among males and 43 suicides among females) were reported in Puerto Rico (Supplementary Table 15). The suicide rate for males was 5.2 times the rate for females (13.0 versus 2.5 per 100,000 population). Suicide rates were highest among men aged 45–64 years (19.0), followed by men aged ≥65 years (17.8). The majority (96.3%) of all suicide decedents were Hispanic.

#### Method and Location of Injury

Hanging, strangulation, or suffocation was the most commonly used method for suicide among both males (57.4%) and females (41.9%) (Supplementary Table 15). For males, the next most common method of injury was a firearm (22.3%); for females, the next most common method was poisoning (34.9%). The most common location where a suicide took place was a house or apartment, both for males (69.5%) and females (86.0%).

#### Incident Characteristics

Suicide decedents were commonly injured in their own homes (67.5%) (Supplementary Table 15). Emergency medical services responded to the scene in over half of cases (52.1%), and 12.2% of male suicide decedents had recently been released from an institutional setting.

#### Toxicology Results of Decedent

Tests for BAC were conducted for 97.5% of suicide decedents (Supplementary Table 18). Among those with positive results for alcohol (i.e., ethanol [23.1%]), 57.4% had a BAC ≥0.08 g/dL. Other than alcohol, suicide decedents were most often tested for cocaine (97.9%), cannabis (commonly referred to as marijuana; 97.1%), and opioids (97.9%). Results for cocaine, cannabis, and opioids were positive in 12.3%, 10.7%, and 7.7% of decedents tested, respectively.

#### Precipitating Circumstances

Circumstances were identified in 94.2% of suicides (Supplementary Table 19). Overall, a mental health problem was the most common circumstance among suicide decedents, with 50.9% having a current diagnosed mental health problem and 54.9% experiencing a depressed mood at the time of death. Among all suicides, 33.2% of decedents were receiving mental health or substance use treatment at the time of death; 43.8% of decedents had a history of ever being treated for a mental health or substance use problem.

Among males, 52.7% of suicide decedents had current depressed mood and 45.7% had a current diagnosed mental health problem. Depression or dysthymia (73.8%) was most often the mental health diagnosis experienced by male suicide decedents with a diagnosed mental health problem, followed by anxiety disorder (15.5%) or schizophrenia (15.5%). More than one third (38.0%) of male suicide decedents had a history of ever being treated for a mental health or substance use problem. Approximately one third (32.1%) of male suicide decedents had a history of expressing suicidal thoughts and plans, and 31.0% had a history of attempting suicide. Other precipitating circumstances for male suicide decedents included physical health problems (15.2%) and alcohol (14.1%) or other substance use problems (12.0%). Nonadherence to mental health or substance use treatment was noted in 7.6% of male suicide decedents.

Among female suicide decedents, 64.3% had a current depressed mood, and 73.8% had a current diagnosed mental health problem. Depression or dysthymia was most often the mental health diagnosis experienced by female suicide decedents who had a diagnosed mental health problem (74.2%). More than two thirds (69.0%) of female decedents had a history of ever being treated for a mental health or substance use problem, and 61.9% were known to have been receiving mental health or substance use treatment at the time of death. Nonadherence to mental health or substance use treatment was noted for 28.6% of female suicide decedents. Half of female suicide (50.0%) decedents had a history of attempting suicide.

## Discussion

In 2023, approximately 78,000 persons in the United States died by suicide, homicide, legal intervention, or unintentional firearm injuries, or their deaths were deaths of undetermined intent that might have been due to violence (*1*). NVDRS provides detailed information such as circumstances of and information on specific manners of death, and the data can be used to describe characteristics of specific populations particularly affected by these fatal injuries. These details increase the knowledge base about the circumstances associated with these deaths and can assist public health authorities and their partners in developing and guiding effective, data-driven approaches to violence prevention.

Deaths recorded in NVDRS affect all persons, regardless of sex, age, or race and ethnicity, and suicide and homicide are among the leading causes of death for all age groups ≤65 years, making them an important public health problem. This report summarizes data on deaths that occurred in 2023 in all 50 states, the District of Columbia, and Puerto Rico.

The occurrence of deaths recorded in NVDRS varies greatly across states, the District of Columbia, and Puerto Rico. State-level violent death reporting system data can be queried via WISQARS (*1*). The 47 states with statewide data and the counties covered in California, Florida, Texas, and the District of Columbia represented 90.9% of the U.S. population (*6*) and accounted for 91.4% of violent deaths and suicides in the United States in 2023 (*1*).

NVDRS helps measure the national prevention initiative Healthy People 2030 objectives related to reducing the rate of suicides, homicides, firearm-related deaths, and child abuse and neglect deaths (*10*). Preventing suicide and homicide can prevent future challenges that arise when children witness violence, lose their parents or other loved ones to suicide or homicide, and have other related experiences that are considered adverse childhood experiences; these factors have been consistently linked to a broad array of negative health outcomes (*11*). These injury-related deaths are preventable and reducing deaths in communities is possible with evidence-based approaches (*12*). CDC developed violence and suicide Prevention Resources for Action to assist communities in identifying prevention approaches that are based on the best available evidence. The resources describe strategies, approaches and specific example programs, practices, and policies with evidence to reduce the risk for suicide, community violence, child abuse and neglect, adverse childhood experiences, IPV, and sexual violence (*11*,*13*–*17*). Each Prevention Resource for Action considers the multifaceted and interactive effects of the different levels of social ecology, including individual, relationship, family, school, and community factors that influence violence- and suicide-related outcomes. NVDRS gathers ongoing, systematic, and consistent data on deaths that can be used by prevention experts within their communities to guide planning and implementation and track outcomes of prevention strategies and approaches.

### Suicides

#### Suicide Circumstances

Approximately one third of suicide decedents had a history of suicidal thoughts or plans, and one fifth had disclosed their suicidal intent. Multiple factors contribute to the risk for suicide (*18*), and the findings in this report indicate that intimate partner problems, recent or impending crises, arguments or conflicts, and physical health problems were common precipitating circumstances. Although one of the most commonly identified circumstances was a currently diagnosed mental health problem, approximately one half of suicide decedents did not have a known diagnosed mental health condition at the time of death. Past suicidal behavior and mental health problems are well-documented as important risk factors to emphasize in suicide prevention (*13*,*18*). One fourth of suicide decedents were known to be receiving treatment at the time of death, indicating a gap between those receiving treatment and those who might benefit from, but are not receiving, treatment.

Mental health problems and substance use also often co-occur among suicide victims (*19*). In this analysis, alcohol use, especially alcohol use in excess of the legal limit (i.e., BAC ≥0.08 g/dL), was frequently observed among suicide decedents who were tested for substances. Although examination of antemortem versus postmortem BAC results was beyond the scope of this report, a substantial proportion of the toxicology testing for this report’s suicide decedents was likely performed postmortem (e.g., the decedent did not survive long enough to receive antemortem testing at a hospital). Other research examining antemortem and postmortem BACs among fatally injured persons has found that postmortem BACs underestimate alcohol involvement (*20*), which suggests that this report’s findings of BACs in excess of the legal limit (64.7% of those testing positive for alcohol) might have been conservative. Alcohol use is a strong predictor of suicidal behavior (*19*,*21*,*22*). Alcohol use can cause disinhibition, increase negative feelings related to oneself or others, lead to impulsive behaviors, and might stem from risk factors that are also risk factors for suicide (e.g., depression and adverse childhood experiences) (*19*). In addition, positive toxicology results for opioids (illegal or prescription) were reported in one fifth of suicide decedents tested for these substances. Previous research has suggested that chronic pain, which might lead to opioid use, might be a contributor to suicide (*23*,*24*). Important activities to address the opioid overdose epidemic include expanding naloxone availability and access to treatment with medications for opioid use disorder, enhancing public health and public safety partnerships, addressing prescription opioid misuse, and maximizing the ability of health systems to link persons who use drugs to services (*25*–*28*).

Another factor that contributes to suicide risk is access to lethal means (e.g., firearms) among persons at risk for suicide (*13*,*29*). Firearms were the most common method used in suicides in the 50 U.S. states and District of Columbia, accounting for approximately one half of the deaths by suicide in this analysis. Because certain lethal means (e.g., firearms) have high case-fatality rates, they confer limited opportunity for immediate life-saving response or intervention (*30*). Males and older adults are more likely than females and younger adults, respectively, to die by firearm suicide (*31*,*32*). This analysis found that suicide rates were highest among males and adults aged ≥75 years. Creating protective environments by reducing access to lethal means among persons at risk can be an effective strategy to prevent suicide (*13*).

#### Demographics

Suicides comprised the majority of deaths collected in NVDRS and occurred at higher rates among males than among females and, in the 50 states and the District of Columbia, occurred at higher rates among AI/AN, White, and NH/PI persons compared with persons of other racial and ethnic groups. Males face a heightened risk for suicide compared with females, particularly older adult males. Specific risk factors contributing to suicide among males include living in rural areas, access to lethal means, alcohol and drug use, relationship breakdown, depression, and lower levels of education (*33*,*34*). These factors often overlap and are shaped by broader social and psychological challenges (*33*,*34*). In addition, the findings in this report regarding suicide rates experienced by AI/AN persons in particular warrant attention to the contextual factors that might contribute to barriers to accessing mental health care, exposure to the suicide of a friend or family member as a contributing factor to a person’s own death by suicide, relationship problems, and alcohol and substance use (*35*).

#### Suicide Prevention Strategies

CDC’s Suicide Prevention Resource for Action identifies the following seven strategies for reducing suicide and suicidal behaviors: 1) strengthen economic supports, 2) create protective environments, 3) improve access and delivery of suicide care, 4) promote healthy connections, 5) teach coping and problem-solving skills, 6) identify and support persons at risk, and 7) lessen harms and prevent future risk (*13*). These strategies support the goals and objectives of the National Strategy for Suicide Prevention, a comprehensive national agenda for suicide prevention (*36*). CDC’s Suicide Prevention Resource for Action includes examples of specific approaches that communities can implement to use each strategy (*13*). CDC’s Comprehensive Suicide Prevention Program supports programs around the country to implement and evaluate a comprehensive public health approach to suicide prevention (*37*); the program focuses on populations disproportionately affected by suicide, such as veterans, tribal populations, rural communities, sexual minorities, and young adults, with a goal of reducing suicide and suicidal behaviors by 10% among these groups. Programs are encouraged to use NVDRS data to guide prevention efforts for each of these populations.

### Homicides

#### Homicides of Infants and Children and Prevention Strategies

Although homicide rates for children varied across age groups, infants (aged <1 year) experienced a higher homicide rate compared with children and adolescents aged 1–14 years. Certain studies have found the highest risk for newborn and infant homicide is on the day of birth (*38*,*39*). Risk starts in infancy and continues throughout childhood, highlighting the need to prioritize strategies focused on the prevention of child abuse and neglect and interventions to reduce risk for morbidity and mortality (*16*). Child abuse and neglect often are associated with immediate physical injuries, emotional and psychological problems, involvement in risky health behaviors later in life, and a wide range of broader physical health challenges and long-term health consequences (*16*).

#### Demographics

Racial and ethnic minority groups experience high rates of violent injury and homicide, particularly among youths and young adults. In the United States, among both males and females, AI/AN and Black persons experienced the highest rates of homicide. In Puerto Rico, the homicide rate was double the suicide rate, and male victims, who predominantly were Hispanic, experienced homicide rates nearly three times the homicide rates experienced by Hispanic males in the 50 states and the District of Columbia. In conjunction with other data sources, NVDRS data can be used to help states and jurisdictions identify and address salient risk factors related to violence at the neighborhood and community levels, which might guide strategies aimed at reducing violence at the population level (*40*). CDC’s Community Violence Resource for Action outlines multiple programs and approaches at the community and societal levels, such as street outreach programs, environmental design activities supporting safe spaces, and policies that strengthen economic stability (*15*). Evaluations of these programs and policies have confirmed the value of using these types of approaches to reduce the risk for violence and promote protective community environments (*15*).

#### IPV-Related Homicides and Prevention Strategies

Homicides of males were most often precipitated by an argument or conflict or occurred during the enactment of a crime. In contrast, nearly half of homicides of females were IPV-related, and a current or former spouse or intimate partner was the identified suspect for over half of female homicide victims with known suspects. CDC’s Intimate Partner Violence Prevention Resource for Action outlines multiple strategies, approaches, and example programs and policies to prevent IPV and to decrease harms (*17*). Strategies and approaches to prevent and reduce IPV might occur across different levels of the social ecology, such as engaging men and boys as allies; disrupting developmental pathways toward IPV; creating protective school, workplace, and neighborhood environments; teaching youths about safe and healthy relationships; empowering bystanders; and strengthening economic supports for families.

### Other Manners of Death

#### Legal Intervention Deaths

NVDRS collects more complete information on legal intervention deaths than any other existing data sources in the United States (*41*). The rate of legal intervention death was highest among AI/AN males (4.2 times higher than the rate for White males), and the rate among Black males was 2.8 times that of their White male counterparts, a finding consistent with previous studies (*42*,*43*).

#### Unintentional Firearm Injury Deaths

Studies using NVDRS data have expanded understanding of unintentional firearm injury deaths, including details about victims and shooters. In this report, approximately half of unintentional firearm injury deaths were self-inflicted; however, approximately one third were inflicted by another person. These deaths often occurred while the shooter was playing with the firearm, unintentionally pulling the trigger of the firearm, showing the firearm to others, or thinking the firearm was unloaded. These circumstances are concerning, particularly when a child is the shooter, the victim, or both. These findings underscore the importance of secure firearm storage and safe-handling practices to prevent unintentional injuries among children and adolescents (*44*), which previous research has shown to have protective effects against firearm-related injury and death (*45*).

#### Deaths of Undetermined Intent

Poisoning was the most common method of injury in approximately one half of deaths of undetermined intent described in this report. Among those tested for substances, a notable proportion of decedents had positive results for antidepressants, cocaine, or opioids (illegal or prescription) at the time of death. Research has demonstrated the challenges and variations in the classification of undetermined intent, particularly those resulting from poisoning, by coroners and medical examiners in the United States (*46*,*47*). These variations can be influenced by the definition of deaths of undetermined intent; the impact of decentralized medicolegal practices; differences in death investigation practices, training, and philosophies; and subjectivity in categorizing deaths (*47*). These findings underscore the importance of careful examination and recognition of the complexity involved in classifying and preventing deaths of undetermined intent.

## Limitations

The findings in this report are subject to at least five limitations, many of which have been described elsewhere (*42*). First, NVDRS data are not yet nationally representative. Second, the availability, completeness, and timeliness of data depend on partnerships between state and jurisdiction violent death reporting system programs and data providers; states and jurisdictions with centralized systems typically have better data availability. Third, toxicology testing is not consistently performed or collected for all decedents or alcohol or drug categories. Fourth, abstractors are limited to the data included in the investigative reports they receive, and data might be less readily available in ongoing investigations. Finally, medical and mental health information often is not included directly from medical records but from coroner or medical examiner records and the decedent’s family members and friends. Therefore, the completeness and accuracy of this information are limited to the knowledge of the informant.

## Using Violent Death Reporting System Data for Action

State and jurisdiction violent death reporting system data have supported jurisdictions in identifying populations disproportionately affected by violence and suicide to develop targeted prevention strategies. For example, in Maryland, where suicide is a leading cause of death among youths (*1*), the Maryland Behavioral Health Administration partnered with the Maryland VDRS to analyze suicide patterns among men under age 30 (*48*). These analyses are now guiding statewide suicide prevention efforts. Veterans represent another population disproportionately impacted by suicide (*49*) and several violent death reporting system programs have supported data-driven prevention initiatives focused on this group. For example, the Michigan Veterans Affairs Agency used Michigan VDRS data to determine that many suicides among current and former military personnel occurred in natural areas. On the basis of this finding, CDC partnered with the Department of Natural Resources to display the 988 Suicide and Crisis Lifeline on hunting and fishing licenses issued to resident active-duty and 100% disabled military veterans. In Connecticut, the Capital Region & Eastern Connecticut affiliate of the Easterseals used data from the Connecticut VDRS to identify demographic suicide prevention service gaps and to secure funding from the Department of Veterans Affairs for expanded veteran mental health and suicide prevention services (*50*).

## Future Directions

As a flexible web-based system, NVDRS continues to innovate and participate in data modernization activities. In 2025, the NVDRS rapid reporting feature was added to the NVDRS web-based system to facilitate optional provisional data reporting at the state and jurisdictional level, improving data timeliness by up to 1 year to more rapidly guide prevention efforts (*51*).

This report summarizes data on NVDRS deaths that occurred in 2023 and is the second consecutive year that data from all 50 NVDRS states, the District of Columbia, and Puerto Rico met inclusion criteria. However, data from California, Florida, and Texas still represent a subset of counties as these states move toward statewide data collection. To achieve full national representation, NVDRS aims to include data for all counties from participating states in future reports.

## Conclusion

Public health surveillance is the foundation for public health practice (*2*). Monitoring the prevalence of violence- and suicide-related fatal injuries, defining priorities, and guiding prevention activities are essential parts of public health surveillance. NVDRS gives communities the violent death information they need for prevention planning and enables national assessment of the need for and impact of violence and suicide prevention strategies.

## Footnotes

* Frequencies and rates of deaths included in this report differ slightly from the frequencies and rates of deaths reported by CDC’s WISQARS, which excludes nonresident deaths (i.e., occurrent deaths) that occur in participating states and the District of Columbia. NVDRS tracks both resident and occurrent deaths in the overall dataset, and the numbers in this report reflect both. VDRS programs are expected to collect information on deaths among their residents and fatal injuries occurring within their borders irrespective of the decedent’s residence status. The state of injury occurrence is responsible for collecting the information. By making this differentiation of responsibility, duplicate reporting in NVDRS is avoided.

† To be included in NVDRS, deaths of undetermined intent must have evidence of the possibility that the intent was purposeful, including use of a weapon or other evidence that force was used to inflict the injury. Most commonly, the coroner or medical examiner is unsure whether the death was a suicide or unintentional.

§ 45 C.F.R. part 46.102(l)(2), 21 C.F.R. part 56; 42 U.S.C. Sect. 241(d); 5 U.S.C. Sect. 552a; 44 U.S.C. Sect. 3501 et seq.
